# COVID-19 in low-tolerance border quarantine systems: Impact of the Delta variant of SARS-CoV-2

**DOI:** 10.1126/sciadv.abm3624

**Published:** 2022-04-08

**Authors:** Cameron Zachreson, Freya M. Shearer, David J. Price, Michael J. Lydeamore, Jodie McVernon, James McCaw, Nicholas Geard

**Affiliations:** 1 School of Computing and Information Systems, The University of Melbourne, Parkville, Victoria, Australia.; 2 Melbourne School of Population and Global Health, The University of Melbourne, Carlton, Victoria, Australia.; 3 Department of Infectious Diseases, The University of Melbourne at the Peter Doherty Institute for Infection and Immunity, Melbourne, Victoria, Australia.; 4 Department of Econometrics and Business Statistics, Monash University, Clayton, Victoria, Australia.; 5 Victorian Infectious Diseases Laboratory Epidemiology Unit, Royal Melbourne Hospital at The Peter Doherty Institute for Infection and Immunity, Melbourne, Victoria, Australia.; 6 School of Mathematics and Statistics, The University of Melbourne, Parkville, Victoria, Australia.

## Abstract

In controlling transmission of coronavirus disease 2019 (COVID-19), the effectiveness of border quarantine strategies is a key concern for jurisdictions in which the local prevalence of disease and immunity is low. In settings like this such as China, Australia, and New Zealand, rare outbreak events can lead to escalating epidemics and trigger the imposition of large-scale lockdown policies. Here, we develop and apply an individual-based model of COVID-19 to simulate case importation from managed quarantine under various vaccination scenarios. We then use the output of the individual-based model as input to a branching process model to assess community transmission risk. For parameters corresponding to the Delta variant, our results demonstrate that vaccination effectively counteracts the pathogen’s increased infectiousness. To prevent outbreaks, heightened vaccination in border quarantine systems must be combined with mass vaccination. The ultimate success of these programs will depend sensitively on the efficacy of vaccines against viral transmission.

## Teaser

Vaccines against COVID-19 can make quarantine safer, but what happens if a new variant emerges?

## INTRODUCTION

Mitigation of pandemics requires a continuous analysis of risk trade-offs to respond proportionately and efficiently. Border quarantine systems are designed to allow travel between jurisdictions while limiting the risk of disease transmission between them. Rigorously limiting disease transmission between regions is appropriate when large differences exist in pathogen prevalence. While strict border measures are effective at preventing disease incursions, they are also costly to operate and reduce international travel to a trickle. Given the enormous economic and social costs associated with the international travel restrictions that come with stringent border quarantine policies, such systems should only be used when they can prevent a catastrophic public health crisis (*1*, *2*).

During the coronavirus disease 2019 (COVID-19) pandemic, border quarantine strategies have been implemented in most parts of the world in various forms (*3*, *4*). In regions with tightly controlled borders, screening of international travelers has provided an effective means of limiting the importation rate of individuals infected with severe acute respiratory syndrome coronavirus 2 (SARS-CoV-2) (*5*–*7*). This has facilitated the success of outbreak control strategies relying on targeted test-trace-isolate-quarantine (TTIQ) responses. This combination of approaches has been largely successful in preventing widespread epidemics of COVID-19 in countries such as China, New Zealand, and Australia (*8*–*11*). In Australia, this has meant that many citizens abroad at the outset of the pandemic have been stranded overseas because of quarantine capacity constraints. It has also stressed the higher education and tourism sectors and devastated the airline industry (*12*–*14*). The design of a quarantine system needs to balance the benefits of reducing the risk of importation against the associated costs. To assess this trade-off, analytical frameworks must incorporate emerging evidence about pathogen characteristics to provide accurate estimates of the risk associated with alternative quarantine strategies.

Two potential factors motivating a reevaluation of quarantine stringency include changes in the properties of a pathogen and changes in what is deemed an acceptable level of breach risk. In the case of COVID-19, the development and rollout of effective vaccines provided an opportunity for countries that had previously maintained stringent border controls to contemplate a future in which these measures could be relaxed.

However, the emergence of the Delta variant of SARS-CoV-2 has produced the need to evaluate risk trade-offs in the context of a virus exhibiting higher transmissibility, higher clinical severity, and against which existing vaccines are less effective (*15*–*22*). This reevaluation of risk is relevant also to the Omicron variant, which has demonstrated a substantially higher capacity to infect vaccinated individuals and those previously infected with different variants (*23*). In this shifting context driven by the emergence of new variants and the changing availability of various types of vaccines and booster programs, the role of vaccination and the acceptable risk of border quarantine breach events must be reevaluated. The emergence of new variants with broadly different disease characteristics can be viewed as the onset of a new pandemic. Therefore, risk trade-offs and mitigation measures need to be assessed on the basis of up-to-date information. In this work, we evaluate the efficacy of border quarantine systems as a function of the following pathogen characteristics: (i) transmissibility and (ii) efficacy of vaccines against transmission (the combination of efficacy against infection and efficacy against transmission from breakthrough cases).

As our primary purpose is to estimate reduction in transmission risk, we focus on pathogen and vaccine characteristics associated with transmission. The efficacy of vaccines for preventing infection and onward transmission is a primary consideration when designing modified border quarantine pathways for vaccinated travelers, which is a widely adopted framework as of September 2021 (*24*). We note here that immune evasion of newly emerged variants capable of reinfecting those who have previously recovered is likely to correspond also to some level of evasion of vaccine-induced immunity and, therefore, to lower values of vaccine efficacy. However, we do not explicitly incorporate preexisting immunity imparted through past infection into our model’s initial conditions, so the detailed ramifications of immune evasion are beyond the scope of the present analysis.

**Fig. 1.**
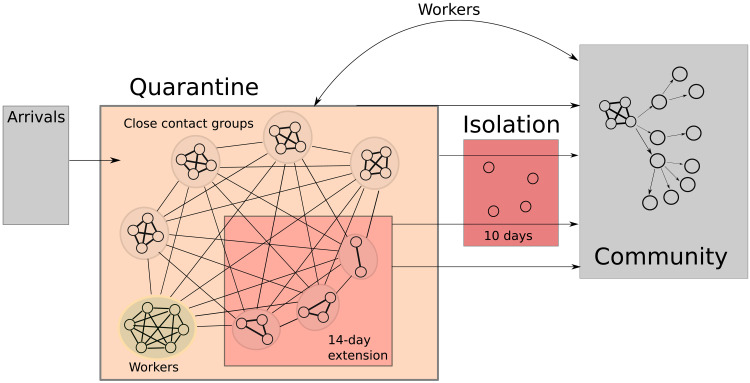
Schematic of the quarantine system model. Arrivals enter quarantine in groups of four close contacts. These groups are in weak contact with one another and with the workforce. If a case is detected, the infected individual is placed into a 10-day isolation, and their contacts are placed into a 14-day quarantine extension. Travelers in extended quarantine are still in contact with the workforce and with other close contact groups within the facility. A separate branching process model is used to evaluate the potential for outbreaks in the community based on the breach event statistics produced by the quarantine system model.

**Fig. 2.**
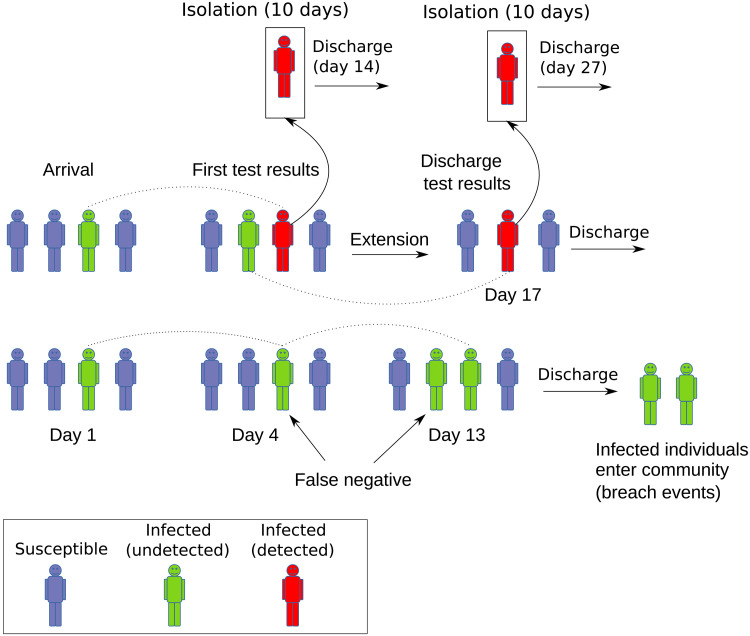
Schematic of quarantine event sequence. Arriving travelers enter quarantine where they are subject to testing, isolation of detected infections, extension of quarantine for close contacts (i.e., family groups), and eventual discharge from quarantine. False-negative discharge tests lead to infected individuals entering the community. Tests are taken on days 3 and 12, with results on days 4 and 13. Isolation lasts for 10 days, after which the patient is discharged from quarantine. Quarantine extension lasts for 14 days and may be followed or interrupted by a 10-day isolation period, but multiple extensions are not implemented.

We simulate border quarantine systems designed based on the recommendations of the World Health Organization (WHO) for countries that choose to quarantine all international arrivals (*25*). Our model includes a 14-day minimum stay, a testing regime (taken on days 3 and 12), and a response strategy that isolates confirmed cases and their contacts from the other travelers in quarantine (Figs. 1 and 2). We assume that transmission within the quarantine system is mitigated substantially through infection control measures (we explore the relaxation of this assumption in our sensitivity analysis). With respect to disease surveillance in the quarantine workforce, our model assumes that staff attending to the quarantine facility are tested on each day they visit the site, but not on days off. Therefore, our model can account for the effects of intermittent work schedules on the capacity to detect transmission between infected travelers and workers. The chosen model reproduces general features adopted by the Chinese, Australian, and New Zealand border quarantine systems. We note that these assumptions do not apply in scenarios where logistical or economic constraints limit the capacity to separate groups of quarantined travelers and where the personal protective equipment required to protect workers from exposure is not available.

We examine the performance of the quarantine system over a range of vaccine efficacy levels and reproductive ratios, relative to an unvaccinated baseline condition (0% vaccine efficacy). We then investigate how the risk of outbreaks seeded by quarantine breach events changes with the emergence of a more transmissible strain (i.e., the Delta variant of SARS-CoV-2). For our outbreak scenarios, we examine the effect of varying levels of population-wide vaccination coverage. This model and the ensemble of results presented here can help guide adaptation of border quarantine measures as the virus evolves, vaccination coverage increases, and vaccine efficacy changes.

## RESULTS

### Summary

Our results demonstrate how the quarantine system would perform under different conditions of vaccine efficacy and viral transmissibility under fixed assumptions with respect to details such as test schedules and quarantine duration (see the Supplementary Materials). We quantify this in terms of the breach events produced under each set of conditions. Breach events occur when an infected traveler or quarantine worker comes into contact with the general population. First, we examine the force of infection produced by breach events, which serves as a measure of quarantine system performance independent of the community in which it is embedded. Then, we examine the potential for outbreaks caused by breach events, taking into account the level of vaccination coverage in the general population. We examine the sensitivity of our results to several of our main assumptions including (i) the disease incubation period, (ii) the decomposition of vaccine efficacy into different components, (iii) the proportion of cases expressing symptoms, and (iv) the effectiveness of infection control measures within quarantine systems.

### Quarantine system breach risk

The quarantine system simulator produces a line list of “breach events,” each of which corresponds to an infected individual exposing the community after either staying in quarantine or working there. For each of these events, we estimate the secondary cases in the community as β*_i_* (the instantaneous rate of infection integrated over the community exposure period; see Methods). The value of β*_i_* corresponds to the expected number of secondary cases from breach event *i*, assuming no prior immunity- or vaccine-induced resistance to infection in the wider population. The sum of these individual community secondary case numbers over all simulated breach events gives the total expected number of community cases over the entire simulation, which is a useful relative measure of system efficacy, that we label β_tot_.

**Fig. 3.**
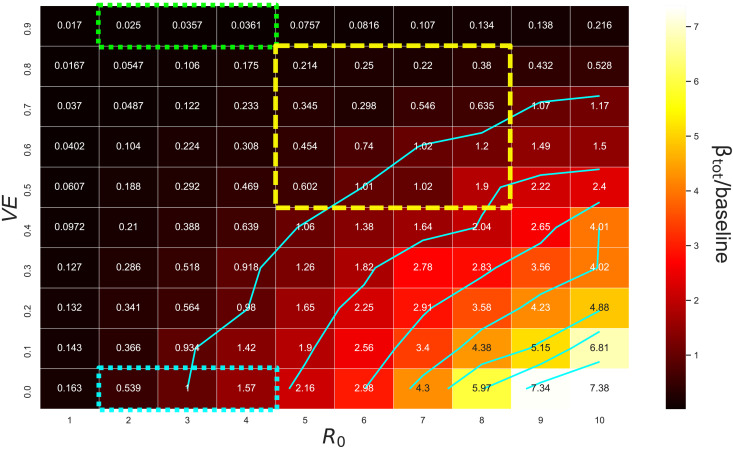
Integrated force of infection relative to baseline from simulated quarantine breach events. The heatmap and contour demonstrates how the relative force of infection produced by quarantine breach events scales with vaccine efficacy (*VE*) and the basic reproductive ratio of the virus (*R*_0_). In these simulations, all incoming arrivals and quarantine workers are vaccinated, with susceptibility to infection reduced by the factor indicated by *VE* (i.e., *VE* = *V*_I_, *V*_T_ = 0). The dotted blue box represents plausible values for the baseline condition when the ancestral lineage and Alpha variant of SARS-CoV-2 were dominant and no vaccines were available. The green dotted box represents the scenarios corresponding to vaccinated quarantine pathways before the emergence of the Delta variant. The yellow dashed box covers a range of values plausible for Delta variant scenarios.

Interpreting β_tot_ as a measure of outbreak potential under each set of conditions, the heatmap in Fig. 3 illustrates how vaccine efficacy must increase to offset the rise in outbreak potential produced by increases in *R*_0_. From baseline conditions (*VE* = 0%, *R*_0_ = 3), following the outermost contour delineated in Fig. 3 illustrates that vaccine efficacy must exceed 60% in order for baseline risk levels to be maintained for an *R*_0_ of 6 and must exceed 70% for *R*_0_ = 8. The required *VE* levels saturate for high values of *R*_0_, with efficacy of 70 to 80% sufficient to maintain baseline risk levels even for *R*_0_ = 10. This saturation occurs because transmission within the quarantine environment is partially constrained by the grouping of arrivals into small cohorts (i.e., family units). This constraint on transmission is contingent upon substantial reduction of exposure risk outside of close contact groups, representative of stringent infection control measures within a facility.

### Baseline scenario, *R*_0_ = 3, *VE* = 0

To align our model of the baseline scenario to observed pre-Delta breach statistics for Australia and New Zealand, we used a basic reproductive ratio *R*_0_ = 3 and assume no vaccination (*VE* = 0). We further assume that the prevalence of infection among arrivals is 1%, which corresponds approximately to arrival prevalence levels observed in Australia and New Zealand (*26*) (note that this rough estimate was confirmed through consultation with Australian state health departments). Breach events can be caused by infected workers or travelers, and we assume that, because of frequent testing, worker-related breach events are always observed. However, traveler-related breach events may go unobserved in the absence of follow-up testing after leaving quarantine. In Australia, there has been no requirement for follow-up testing after quarantine discharge. Therefore, in aligning our model results to observations, we assume that breach events associated with travelers would be observed if either (i) the infected individual eventually expresses symptoms outside of quarantine or (ii) the individual infects secondary cases, leading to a local transmission cluster and outbreak response. For the purposes of comparing our model output to observations, we approximate the probability of (ii) as *p*_obs_ = 1 − Poisson(0, β*_i_*). This approximation assumes no attenuation of the infection rate due to public health measures that may have been ongoing at the time of a breach and is therefore a conservative overestimate. To calculate the number of observed breach events from a simulated line list, we evaluate conditions (i) and (ii) for each traveler-related breach. By repeating this evaluation many times, a distribution of possible observed breach numbers can be generated for comparison with real-world data.

This analysis of the baseline scenario line list produces an average of 579 traveler-related breach events (SD σ = 13.8, *n* = 1000 repeats) and 423 worker-related breach events. These absolute counts come from a total of 7.07 × 10^6^ simulated arrivals, for a breach rate of 1.4 × 10^−4^ breach events per arrival. This comparison is sensitive to choice of ascertainment probability. For example, if ongoing public health measures are assumed to bring the community exposure rate down by a factor of 2 [i.e., *p*_obs_ = 1 − Poisson(0, β*_i_*/2)], a mean of 406 traveler-related breach events (SD σ = 13.4, *n* = 1000 repeats) is produced, for a total breach rate of 1.17 × 10^−4^ breach events per arrival.

The breach rate estimated for Australia between 31 March 2020 and 1 May 2021 is approximately 6 × 10^−5^ breach events per arriving traveler. This can be confirmed from publicly available media reports (see, e.g., breach statistics posted to covidlive.com.au; a copy of the accessed data is included in the Supplementary Materials). We verified these overall rates in consultation with Australian state health departments. About half of these events were associated with workers, and half with travelers (this breakdown was ascertained through analysis of detailed reports from Australian state health departments and is not publicly available). Overall, our baseline quarantine system model produces “observable” breach rates that are higher than those computed from public reports by approximately a factor of 2. The model provides a close estimate of the relative rate of breach events associated with workers and travelers, respectively.

On the basis of the summary data aggregated to media reports and posted on covidlive.com.au, 14 quarantine breach events occurred over the 12-month period from April 2020 to March 2021, out of which 9 were associated with onward transmission. We validate our combined quarantine system and community transmission branching process model against this overall outbreak rate by sampling the breach event line list produced by the quarantine model at a rate equivalent to 6000 arrivals per week. This rate of travelers passing through quarantine was chosen to match the average number of international arrivals per week, rounded to the nearest thousand (*27*). The branching process model then assumes a community reproductive ratio of 1.5, reduced by a factor of 2 to account for ongoing public health and social measures in the Australian context. Defining outbreaks as breach events producing more than five onward cases, the model produces on average 20 (SD σ = 4.5) outbreaks per year, or 6.4 × 10^−5^ outbreaks per arriving traveler. Comparing this final outbreak rate to the rate of observable breaches (1.17 × 10^−4^) produced by the quarantine system model, our combined simulation method estimates that approximately half of observable breaches become outbreaks involving onward transmission. This ratio (0.55 outbreaks per observable breach) is broadly consistent with the data aggregated from media reports (0.64 outbreaks per observed breach), while the absolute rate of outbreaks simulated is high by a factor of 2.22 (9 observed and 20 simulated). With the unknown influence of observation bias, a high level of uncertainty in important parameters such as the effective reproductive ratio in the community, and the true prevalence of infection in arriving travelers, we interpret this as an acceptable degree of alignment to real-world observational data.

While our model produces breach and outbreak statistics on the same order of magnitude as publicly reported event data, our aim is not to precisely reproduce historical observations. Jurisdictional quarantine policies and overall epidemic situations changed markedly over the pre-Delta period, and the outbreak statistics to which we compare our model are prone to strong observation bias (amalgamated from media reports). The comparison to Australian breach statistics given here is provided to explain how our models can be calibrated, and to give an indication of the level of achievable realism, given available data.

As noted by Grout *et al.* (*26*), the observed rate of breach events can vary substantially between regions, even when similar quarantine arrangements are in place. Even if we assume perfect disease surveillance and reporting, the small number of recorded events makes high-confidence calibration to “ground-truth” breach rates infeasible. Therefore, the present work focuses on evaluating the performance of a uniform quarantine policy based on globally accepted guidelines, applied over a wide range of viral transmissibility, vaccine efficacy, and vaccine coverage parameter values, to provide useful relative comparisons between scenarios.

### Community outbreak risk

Next, we simulate outbreaks based on the distribution of quarantine breach events produced by the quarantine model. For these scenarios, we investigate a subset of *VE* and *R*_0_ combinations corresponding to plausible values for the Delta variant of COVID-19. We investigated population outbreak characteristics for *VE* ∈ [0.5, 0.6, 0.7, 0.8, 0.9] and for *R*_0_ ∈ [5, 6, 7, 8, 9, 10] while varying mass vaccination coverage levels. In our outbreak simulations, we implement population vaccination as a static coverage level (proportion vaccinated) that we independently vary, not as dynamically increasing coverage. Furthermore, for each outbreak scenario, we assume that all vaccinated individuals have been given a vaccine with the same efficacy and no waning of immunity has occurred. The results should therefore be interpreted as “snapshots” of risk, for each given set of conditions.

For the branching process scenarios, a fixed traveler arrival rate of 3000 per week was assumed, with an infection prevalence of 10%. This differed from the inflow assumptions of the quarantine model (50 travelers per week, 1% infection prevalence), so a scaling factor was used to linearly adjust the breach rate produced by the quarantine model (see methods in the Supplementary Materials). This scaling approximation assumes linear dependence of the quarantine breach rate with incoming arrival infection prevalence of up to 10%, an assumption we verified in the context of the quarantine model.

**Fig. 4.**
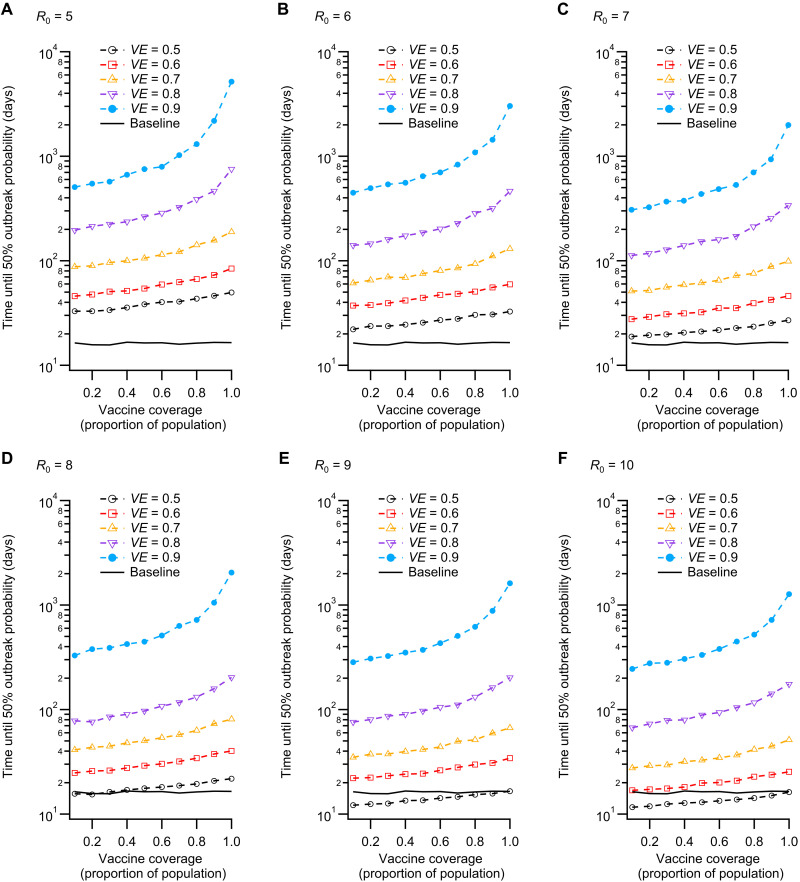
Time until probability of an outbreak in the community reaches 0.5. (**A** to **F**) This set of scenarios corresponds to potential Delta variant parameter combinations. Here, outbreaks are defined as cases ≥5. The vaccine coverage values correspond to the proportion of individuals in the community (outside of quarantine) who are vaccinated. Higher vaccine coverage and efficacy reduce the probability of an outbreak occurring, given a quarantine breach event. Possible threshold behavior is observed for high coverage and efficacy, with vaccine efficacy of 90% producing *t*_50_ values that increase by approximately one order of magnitude as coverage rises from 0.1 to 1.0 (full vaccine coverage). The baseline scenario (black line) shows *t*_50_ with no vaccination (*VE* = 0) and *R*_0_ = 3.

To compare scenarios, we computed the time required to reach a 50% probability of producing a single outbreak (cases ≥5), which we denote *t*_50_. We present *t*_50_ values as a function of the proportion of the population vaccinated (Fig. 4), which demonstrates the effect of mass vaccination on outbreak risk, for each combination of *VE* and *R*_0_. The results illustrate that vaccine efficacy is a crucial determinant of outbreak risk. Even for high coverage, a vaccine with efficacy below 60% is not sufficient to markedly reduce the time required for outbreaks to occur. On the other hand, effects related to herd immunity thresholds in *VE* and coverage are observed for *VE* ≥ 80%, with *t*_50_ increasing rapidly (super-exponentially) after coverage exceeds ≈80%. The effect on *t*_50_ of increasing *VE* from 80% to 90% is marked, even for low coverage, which is a consequence of the high effectiveness of the quarantine system under these conditions. Therefore, our combined quarantine and community transmission models suggest the combined effects of a threshold related to vaccine efficacy in the context of a fully vaccinated quarantine system, coupled with a population coverage threshold for high *VE*. The result is a large increase in the time required for a single outbreak in our community transmission model, with *t*_50_ rising from approximately 15 days (baseline) to the order of 10^3^ days for high coverage and efficacy, over a wide range of *R*_0_ values (as *R*_0_ increases, higher values of *VE* and coverage are required). The sensitivity of outbreak risk to *VE* emphasizes the importance of accurately estimating this crucial parameter (vaccine efficacy against infection and onward transmission).

### Sensitivity analysis: Incubation period

The disease incubation period of the Delta variant of COVID-19 may be shorter than the estimates made for previously dominant lineages. In our sensitivity analysis, we compare a 5.5-day incubation period [corresponding to the ancestral lineages of COVID-19 (*28*)] to a shorter one (4.4 days), corresponding to possible revised estimates for the Delta variant (*16*). The results of our sensitivity analysis show that shorter incubation periods make the quarantine system more effective because (i) overall infectious periods are shorter, (ii) test sensitivity increases more quickly after infection, and (iii) the shorter delay to symptom expression hastens detection after false-negative arrival tests (fig. S11). These results emphasize that shorter incubation periods can make it easier to detect infections in closed systems like quarantine facilities. This reduces the risk to the community for the same quarantine length of stay (see Methods and the Supplementary Materials for more details of this sensitivity analysis).

### Sensitivity analysis: Vaccine efficacy against transmission from breakthrough infections

Vaccine efficacy can arise from different mechanisms determining how well the vaccine (i) protects susceptible individuals from becoming infected and (ii) reduces the capacity for onward transmission from infections in vaccinated individuals. Different combinations of (i) and (ii) can produce the same value of *VE* in a population with fixed coverage. However, in our outbreak model, the choice of (i) and (ii) can alter the risk associated with breach events for the same *VE*. This is because we typically assume a higher level of vaccine coverage in the quarantine environment (100%) than in the community (varied from 10 to 100%).

In both the quarantine model and the outbreak model, we treat disease transmission as a two-part process first requiring infection (subject to the efficacy term *V*_I_, efficacy against infection) and then requiring onward transmission (subject to the efficacy term *V*_T_, efficacy against onward transmission). The overall efficacy against transmission *VE* is given as

$$\mathrm{VE}=1-(1-V_{T})(1-V_{I})$$ (1)

which means that two extreme interpretations exist: (i) *VE* = *V*_I_, *V*_T_ = 0 and (ii) *V*_T_ = *VE*, *V*_I_ = 0. Our main results use the first extreme (see Methods for a more detailed discussion of this choice). We investigate the second extreme in our sensitivity analysis. This gives the range of results over which any decomposition consistent with Eq. 1 may fall.

The results demonstrated in Fig. 3 assume no effect of vaccination on the capacity for vaccinated individuals who become infected to transmit the virus (*V*_T_ = 0, *V*_I_ = *VE*). The alternate assumption that efficacy against onward transmission is equivalent to the total *VE* represents a plausible upper bound, which we investigated in a sensitivity analysis (*V*_T_ = *VE*, *V*_I_ = 0). The results in fig. S12 demonstrate that under this alternate (optimistic) assumption, the vaccine efficacy required to maintain baseline risk levels falls by about 20%. For example, an *R*_0_ of 6 would require a 40% effective vaccine to maintain baseline outbreak risk, while an *R*_0_ of 8 would require a 50% effective vaccine (see Methods and the Supplementary Materials for more details of this sensitivity analysis). This result is consistent with the intuition that when an infected individual who is vaccinated leaves the quarantine system, it is the vaccine efficacy against onward transmission (*V*_T_) that determines their immediate risk to the community. While *V*_I_ prevents someone in quarantine from getting infected in the first place, it does not prevent an infected individual from transmitting to susceptible, unvaccinated members of the wider community after leaving quarantine.

### Sensitivity analysis: Clinical presentation rate

Because clinical detection is an important aspect of case ascertainment, the fraction of infections that result in symptom expression is a key model parameter. In our main analysis, we used an estimate of *p*_symp_ = 0.67 symptomatic infection probability. In the supporting information, we relax this assumption and show heatmaps of β_tot_ relative to the baseline value (i.e., with *R*_0_ = 3, *VE* = 0, *p*_symp_ = 0.67), for *p*_symp_ = 0, *p*_symp_ = 0.5, and *p*_symp_ = 1.0.

This analysis demonstrates a strong quantitative effect of symptom ascertainment on the efficacy of quarantine systems but does not markedly alter the main results of our study. That is, even if all cases are asymptomatic, vaccine efficacy of approximately 60% is sufficient to maintain baseline risk levels (a shift of about 10%, comparing the positions of the outermost contours of Fig. 3 and fig. S13C). However, we note that the increase in risk associated with lower *VE* and higher *R*_0_ is amplified in this case by approximately a factor of 2. In other words, while the vaccine requirements necessary to maintain baseline risk for higher *R*_0_ are not markedly altered, the consequences of not meeting those requirements are amplified if cases are primarily asymptomatic. This could have operational ramifications if, for example, a viral variant emerged that continued to transmit between vaccinated individuals but produced symptom expression only in the most severe cases.

In addition to the fixed parameter implementations above, we investigated the possibility that vaccines reduce the clinical fraction. To do so, we computed the probability that a case will develop symptoms after incubation as *p*_symp_ = 0.67(1 − *VE*). If *VE* = 0, the clinical fraction is equivalent to the base model and approaches zero as *VE* approaches 100%. As *R*_0_ increases from baseline, this dependency marginally increases the vaccine efficacy required to maintain baseline risk (fig. S13D) but does not produce any qualitative changes to the results shown in Fig. 3.

### Sensitivity analysis: Efficacy of infection control measures

In our model of the quarantine system, we assume that transmission is mitigated substantially between travelers in different groups (to 1%), between travelers and workers (to 1%), and between workers (to 10% of the uncontrolled transmission rate). The mitigation factors chosen for our model were selected in orders of magnitude to approximately match the small amount of case data available from consultation with state health departments. These parameters are therefore mostly unconstrained and represent fairly strong assumptions about the effectiveness of transmission control within quarantine systems. We explore the ramifications of allowing increased levels of transmission within the system by increasing these uniformly by one order of magnitude. This means that the modified transmission strength increases to 10% of the unmitigated rate between travelers in different groups and between travelers and workers. For workers, transmission is unmitigated (100% of the base rate). We note that these choices are somewhat unrealistic, as numerous infection control measures were implemented in the jurisdictions for which we obtained information. However, we explore these scenarios to convey the importance of effective local mitigation measures in environments with known exposure risk.

When these infection control measures are compromised, transmission within the quarantine system can markedly amplify the risk of breach events posed by arriving travelers, if vaccination is not sufficiently effective (fig. S14). Under these circumstances, the vaccine efficacy required to maintain baseline risk levels increases (particularly for lower *R*_0_ values). Furthermore, for high reproductive ratios (*R*_0_ of 8 to 10) and low vaccine efficacy (*VE* below 30%), our model simulated breach risk exceeding baseline by up to two orders of magnitude. These results highlight the extreme importance of infection control within quarantine facilities that adopt the “test and release” strategy typically used in modern systems that rely on transmission mitigation and case isolation.

## DISCUSSION

The vaccines that have been developed against SARS-CoV-2 remain highly effective at preventing severe disease. However, their efficacy against infection has decreased against the Delta variant of the virus, and efficacy may deteriorate further with the continued emergence of new variants (*29*, *30*). In the context of border quarantine, the capacity to limit transmission is the key consideration when determining how best to manage new arrivals, some of whom may be infected (asymptomatic or presymptomatic). This is because the primary purpose of a border quarantine system is to prevent infectious individuals from entering the community. The management of clinical cases within the quarantine system is facilitated by regular surveillance, efficient case detection, and the allocation of medical resources. Therefore, the utility of vaccination within the context of a quarantine system is not equivalent to the utility of mass vaccination in the context of a large outbreak. In large outbreaks, efficacy against clinical severity reduces hospital caseloads and deaths, mitigating the public health burden and human cost, even if transmission continues. However, in modern quarantine systems, the operational goal is to identify and isolate cases to limit transmission and keep the required duration of quarantine to a minimum.

The 14-day minimum stay commonly practiced during the COVID-19 pandemic was implemented because of the long incubation period of the disease and long infectious periods for those who remain asymptomatic. Within 14 days, a case arriving while presymptomatic would be likely to display symptoms, and, secondary transmission notwithstanding, most asymptomatic cases would have recovered. Testing of arrivals through reverse transcription polymerase chain reaction (RT-PCR) accelerates the process of case detection and facilitates earlier management. When these case detection efforts are unsuccessful, transmission within the quarantine environment, at best, leads to extended stay conditions for cases and their contacts. At worst, it leads to the discharge of presymptomatic infectious individuals who receive their exit test early in their infection and do not yet have high enough viral load for case confirmation. Therefore, the primary benefit of vaccination in the context of quarantine facilities is in limiting transmission.

In this context, the increased transmissibility and decreased vaccine efficacy against transmission associated with the Delta variant require a reevaluation of risk. Our results demonstrate that vaccination may allow quarantine systems to remain effective. On the other hand, quarantine requirements for vaccinated travelers must remain stringent because of the increased transmissibility of the virus. To emphasize the implications of this result, had conditions remained consistent with the Alpha variant (*R*_0_ ≈ 3, *VE* ≈ 90%), Fig. 3 indicates that vaccination would have decreased border quarantine breach risk to 3% of baseline. In effect, the vaccinated quarantine pathway would have allowed the number of arrivals to increase by a factor of approximately 30 (assuming sufficient system capacity) while maintaining baseline community exposure levels.

Under the existing circumstances, our analysis suggests that quarantine policies for vaccinated individuals will need to approximate those that were used for unvaccinated cohorts before the emergence of the Delta variant. Unvaccinated cohorts of travelers, on the other hand, will pose a much greater risk of quarantine breach events than they did previously (e.g., increased by a factor of 3 for *R*_0_ = 6; Fig. 3).

Ultimately, the level of stringency in requirements for quarantine of new arrivals should be assessed as a function of the prevalence of viral variants between jurisdictions. Where these relative levels are similar for variants of concern, there is little justification for limitations on travel. However, with the spatially localized emergence of new variants, quarantine systems must be capable of rapidly responding to slow, or eliminate, the global diffusion of variants. This is particularly true for variants with increased transmissibility and clinical severity (of which the Delta variant of SARS-CoV-2 is a primary example).

To summarize, our results demonstrate that, in the context of SARS-CoV-2, border quarantine systems cannot be used to compensate for low levels of community vaccination. For the Delta variant, this is true even when all individuals within the quarantine environment are vaccinated. This is because the Delta variant is more transmissible and likely to produce breakthrough infections in individuals vaccinated against the ancestral lineage. Our findings illustrate a key aspect of the drawn-out global battle to mitigate the public health crisis produced by COVID-19. Just as regions with low community prevalence were becoming confident that international travel could increase for vaccinated individuals, the virus changed to become more transmissible, and to partially avoid vaccine-induced immunity. Our results show that these changes nullified the prospective benefits of vaccination within quarantine systems in terms of international travel volumes. In Australia and New Zealand, the emergence of the Delta variant quickly resulted in ongoing community transmission [in June and August of 2021, respectively (*31*)]. In the context of community transmission, it is difficult to verify the effectiveness of the subsequently introduced requirement for travelers to be vaccinated (*32*). Furthermore, with continuing transmission of the Delta variant in Australia, there was a marked acceleration of the vaccination campaign, which saw the introduction of relaxed quarantine requirements for vaccinated travelers.

Moving forward, the expansion of quarantine systems should focus on preparing for future variants of COVID-19 and, ultimately, for future pandemics with higher clinical severity and infection fatality ratios. COVID-19 has revealed the unprecedented capacity for populations around the world to markedly alter their behavior to prevent disease spread. Quarantine systems amplify the payoff of these population-wide responses by limiting incursions. The coupling of border quarantine measures with elimination strategies buys critical time for the development of vaccines and effective clinical practices.

## METHODS

### Model overview (structure, inputs, and outputs)

We simulate virus transmission, case detection, and isolation response pathways within a single quarantine facility with a capacity of 100 travelers. The facility is staffed by 20 vaccinated workers who have intermittent contact with those in quarantine. Individuals are processed subject to testing and case isolation. The structure of the quarantine environment is generic but captures the main principles typically applied in border quarantine facilities. The output of the quarantine model is used in a separate branching process model to evaluate the potential for outbreaks of community transmission. A schematic of the overall system is shown in Fig. 1.

The population is structured in two distinct groups, one of these comprising workers who staff the facility, and the other comprising the quarantined travelers. Travelers move through the system as indicated in Fig. 2. After arriving in close contact groups of four individuals, travelers remain in the system for 14 days unless an infection is detected within their group. The 14-day minimum stay means that, typically, 50 travelers exit the system each week.

Grouping of travelers in parties of four allows some unmitigated transmission between individuals in close contact, who could be thought of as family groups. However, we do not explicitly simulate the age of individuals, and so our model is not equipped to assess the ramifications of case isolation policies in the context of families. That is, our model as implemented cannot account for situations in which isolation of a case would require the separation of children from their parents or legal guardians. The choice not to fully isolate cases in these situations could result in a higher level of transmission. In addition, young children may not be eligible for vaccination, which is not accounted for in the model as parameterized for this work.

The model used to simulate border quarantine incorporates a detailed description of COVID-19 progression and transmission that captures the following salient features: (i) a lognormally distributed incubation period [mean of approximately 5.5 days (μ = 1.62, σ = 0.418); fig. S8]; (ii) time-varying infectiousness, increasing from the moment of exposure, peaking just before symptom onset, and declining until recovery (fig. S4); (iii) time-varying RT-PCR test sensitivity with a peak before symptom onset followed by a gradual decline (fig. S6); and (iv) an overdispersed secondary case distribution (fig. S2).

These features allow the model to capture two important effects of the quarantine environment. The first of these is the truncation of the naturally overdispersed secondary case distribution due to physical separation of close contact groups. The second is the tendency for false-negative tests to occur during the early stages of infection. Detailed descriptions of disease natural history and test sensitivity models can be found in the Supplementary Materials.

Detection of infection can occur due to either positive RT-PCR tests (conducted on days 3 and 12) or symptom onset. The model assumes that one-third of all cases are asymptomatic (this assumption is relaxed in a sensitivity analysis; see the Supplementary Materials). Asymptomatic cases can only be detected through testing. Symptomatic cases may be detected during the presymptomatic period through RT-PCR. If not, they are detected at the end of their incubation period, when they begin expressing symptoms (all symptomatic individuals are treated as confirmed cases).

Case detection in travelers results in a 10-day isolation period for the case, as well as a 14-day quarantine extension for close contacts with additional tests on days 3 and 12 of the extension period. Subsequent detection within the same group of contacts results in isolation of cases but does not incur additional extensions for the remaining contacts. Therefore, the maximum period for which an individual can remain in the system is 38 days. This would occur if an individual were the close contact of a case detected on day 14 of the initial stay, were detected as a case themselves on the 14th day of their extension period, and were subsequently isolated for 10 additional days. While in quarantine extension, transmission dynamics are not altered. While in isolation, individuals may not transmit infection to any other individuals. When all members of a close contact group are discharged from the system, they are replaced by a new group.

In a simulated quarantine facility, the workforce is composed of 20 individuals who come and go each day. Workers are tested via RT-PCR on each day they attend the site. Each worker attends for 5 days per week and has 2 days off per week. Workers may become infected through contact with quarantined travelers or through contact with infected co-workers. The force of infection applied between travelers and workers is reduced by a factor of 100 to simulate infection control measures (e.g., mask wearing) and limited contact. On the other hand, the force of infection between workers is only reduced by a factor of 10 relative to unmitigated contact. This accounts for infection control, with higher levels of mixing. Because workers are tested frequently, infections are typically detected during the presymptomatic period. Infected workers are replaced with susceptible ones after either detection or recovery. We note that replacement after recovery is not strictly realistic but avoids eventual saturation of the recovered worker population over long simulations.

### Scenarios: Vaccine efficacy and pathogen transmissibility

The scenarios we selected are designed to determine how the risk associated with quarantine breaches is mitigated within a pathway exclusive to fully vaccinated travelers. Such a pathway would be staffed exclusively by vaccinated workers as well, for 100% vaccination coverage within the system. In this context, we examine performance over a wide range of vaccine efficacy and viral transmissibility parameters. Performance is determined relative to a baseline scenario, in which vaccine efficacy (*VE*) is set to 0, and disease transmission parameters are aligned to COVID-19 strains in circulation before the emergence of the Delta variant.

In the quarantine and community transmission model systems, vaccines play three important roles: (i) vaccination of travelers before departure reduces the proportion of infected arrivals by a factor of (1 − *VE*) from the base rate of 1%; (ii) vaccination of workers and travelers limits transmission within quarantine (which reduces the rate of breach events); and (iii) mass vaccination prevents outbreaks in the community when quarantine breach events occur.

For efficacy of vaccination against transmission, we investigate the range between 0 and 90% total efficacy. While the quarantine model treats vaccine efficacy as a combination of efficacy against infection *V*_I_ and efficacy against onward transmission from breakthrough infections *V*_T_, we simplify to a single efficacy parameter for the scenarios investigated here. This is because we assume that all individuals in the system are vaccinated. We note that for scenarios in which only a subset of individuals are vaccinated, this simplifying assumption would need to be relaxed to account for interactions involving combinations of vaccinated and unvaccinated individuals. As noted above, we consider an efficacy of 0% as a baseline, accounting for the levels of incursion risk existing before vaccines became available. Efficacy levels of 80% to 90% are indicative of the conditions existing for the COVID-19 ancestral lineage and Alpha variants previously in circulation (*33*, *34*). Lower efficacy ranges can account for the emergence of variants capable of higher levels of breakthrough infection (such as the Delta variant).

The emergence of viral variants also requires us to investigate a broad range of transmission rates. The transmissibility of the Delta variant has been estimated to be approximately twice that of the ancestral lineages (*35*). On the basis of a well-traced outbreak in China (Guangdong province, May 2021), the basic reproductive ratio for the Delta variant is estimated to be approximately 6 (*17*, *16*). Therefore, to understand the scaling of quarantine system performance with disease transmissibility, we examine a wide range of possibilities from *R*_0_ = 1 to *R*_0_ = 10.

### Model variations: Shorter incubation period

In our baseline model, we sample the viral incubation period (time between infection and symptom onset) as described in studies of the ancestral lineage (*28*). This estimate also influences our model of test sensitivity as a function of time from symptom onset (see the Supplementary Materials) (*36*).

Quarantine systems operating under the test and release framework are primarily designed to prevent individuals infected elsewhere from entering the community. The timing considerations applied to this operational framework (i.e., a 14-day minimum stay) are designed to exceed the disease incubation period. From this perspective, the emergence of variants with incubation periods different from those for which the system was designed can be expected to alter system performance. System performance should, in general, improve for shorter incubation periods.

In the context of COVID-19, recent reports suggest that the Delta variant may have a shorter incubation period than that of the ancestral lineage (*16*). However, other reports using data from the same outbreak indicate that the incubation period of the Delta variant has not changed substantially (*17*). Given the preliminary evidence for a shorter incubation period, we performed a sensitivity analysis of this key parameter (see the Supplementary Materials).

### Model outputs

The quarantine facility model produces a time series of breach events, each of which corresponds to an infected individual (worker or traveler) interacting with the community outside of quarantine. For an infected traveler, this can occur for two main reasons: (i) leaving case isolation while still infectious (i.e., after the 10-day isolation period) or (ii) leaving quarantine after a false-negative test result. It is also possible for an individual to become infected after their day 12 test and be discharged while presymptomatic. The recorded breach event accounts for the number of days over which the individual will remain infectious after leaving quarantine and the integrated force of infection produced by the individual over that period. A detailed breakdown of breach events from each of eight possible pathways through the quarantine system is shown in table S3. For infected workers, recorded breach events account for the time from infection to either detection or recovery, and the integrated force of infection over that period. In general, the community force of infection produced by a breach event associated with case *i* is given as

$$\beta_{i}=\sum_{t\in t_{c}}\beta (t,i)\Delta t$$ (2)

where β*_i_* is the integrated force of infection produced by agent *i* outside of quarantine, *t_c_* represents the set of discrete time points over which individual *i* is infectious in the community, β(*t*, *i*) is the time-dependent force of infection for case *i*, and Δ*t* is the discrete time step used in the simulation (here, Δ*t* = 0.1 days).

Breach events are rare because of the effectiveness of the quarantine system, particularly when those within it are vaccinated at high efficacy. To generate a large number of breach events for use in comparing outbreak statistics between scenarios, each simulation lasts for 10^6^ days. This duration is not meant to reflect the time scale of a real epidemic. Rather, the simulations are designed to generate a large number of events in a way that is computationally efficient, by simulating a small stochastic system that is continuously refreshed with new arrivals, rather than simulating many independent systems on shorter time scales. The system’s internal dynamics occur on the order of days to weeks, so the simulation time frame is longer by five orders of magnitude. This ensures that the statistics drawn from each line list essentially represent sets of independent measurements while still capturing potential correlations caused by multiple generations of transmission within the system.

**Fig. 5.**
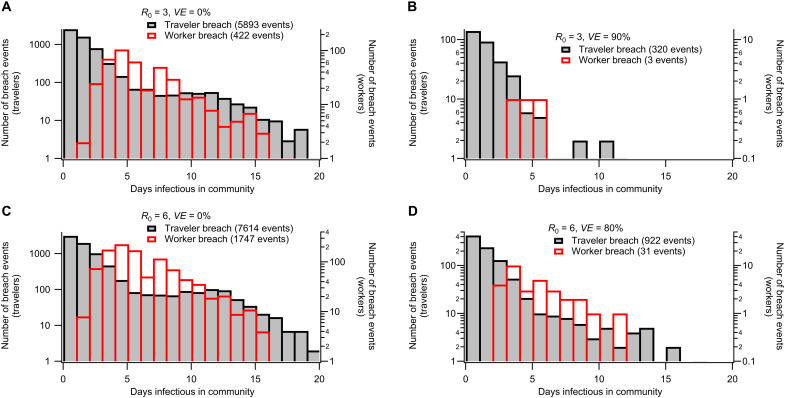
Breach event statistics for selected scenarios. Histograms demonstrate how simulated quarantine breach events are distributed in terms of the number of days an infectious individual exposes the community. The four subplots correspond to illustrative scenarios: (**A**) ancestral strain without vaccination, (**B**) ancestral strain with vaccination, (**C**) Delta strain without vaccination, and (**D**) Delta strain with vaccination (increased transmissibility and decreased vaccine efficacy). Breach event scan occurs from infected travelers being discharged from quarantine while still infectious. They can also occur due to infected workers who are not detected while presymptomatic or asymptomatic due to false-negative RT-PCR screening tests. The distribution of days infectious in the community differs qualitatively for traveler- or worker-related breach events. Each subfigure corresponds to a different combination of reproductive ratio *R*_0_ and vaccine efficacy *VE*. Shaded black bars correspond to traveler-related breach events, while open red bars correspond to worker-related breach events.

Recall that workers are tested daily so that infections are typically detected during the presymptomatic period. Infected workers are replaced after either detection or recovery (the latter avoids eventual saturation of the recovered worker population over long simulations). The different conditions for breach events involving travelers and workers produce qualitatively different breach statistics that depend also on vaccine efficacy and *R*_0_ (Fig. 5). More details of the quarantine simulation model can be found in the Supplementary Materials.

We evaluated the effectiveness of the quarantine system by examining the integrated force of infection introduced into the community due to breach events. This value is computed as

$$\beta_{\text{tot}}=\sum_{i=1}^{n}\beta_{i}$$ (3)

where *n* is the total number of breach events simulated in a given scenario, and β*_i_* is the force of infection in the community produced by breach *i*. Because all other model parameters are held constant (see Methods), β_tot_ is a useful representation of the relative performance of the system for different combinations of *VE* and *R*_0_. In Fig. 3, β_tot_ computed for each set of parameters is shown relative to the value computed for the baseline scenario with *R*_0_ = 3 and *VE* = 0%.

Here, we assume that breakthrough cases are just as contagious as infections in unvaccinated individuals. That is, we assume that the vaccine acts primarily to protect those immunized from infection (*V*_T_ = 0, *V*_I_ = *VE*). While there is evidence for reduced periods of viral shedding in vaccinated individuals, peak viral loads appear to be similar (*37*). Therefore, we have presented the results based on a conservative assumption that can be relaxed or modified as new evidence emerges (see the Supplementary Materials for a sensitivity analysis given an alternate assumption that *V*_T_ = *VE* and *V*_I_ = 0).

The influence of the quarantine system on outbreak risk was computed by using the distribution of breach events produced by the quarantine simulation to sample seeding events for the branching process model. In these outbreak scenarios, the level of population-wide vaccination is varied, assuming a negligible level of infection-acquired immunity. The results of the branching process model are used to estimate the probability of a community outbreak, given a fixed volume of travelers and a set infection prevalence. This produces an absolute outbreak risk that we express as the time until the probability of a transmission cluster containing more than five cases exceeds 50% (*t*_50_). These values should be interpreted as a means of comparing alternate scenarios, given fixed quantities of incoming arrivals (see the Supplementary Materials for details of the branching process model).

## Supplementary Materials

### This PDF file includes:

Sections S1 to S7 Figs. S1 to S14 Tables S1 to S3

## Notes

View/request a protocol for this paper from *Bio-protocol*.
