# Supplementary material for: COVID-19 in low-tolerance border quarantine systems: Impact of the Delta variant of SARS-CoV-2
Source: Sci Adv. 2022 Apr 8;8(14):eabm3624. doi: 10.1126/sciadv.abm3624 (PMC8993115; doi:10.1126/sciadv.abm3624)
Supplement: Supplementary file 1 — Sections S1 to S7 Figs. S1 to S14 Tables S1 to S3 [file sciadv.abm3624_sm.pdf]

Science Advances
AAAS

Supplementary Materials for

COVID-19 in low-tolerance border quarantine systems: Impact of the Delta variant of SARS-CoV-2

Cameron Zachreson*, Freya M. Shearer, David J. Price, Michael J. Lydeamore, Jodie McVernon, James McCaw, Nicholas Geard

*Corresponding author. Email: cameron.zachreson@unimelb.edu.au

Published 8 April 2022, *Sci. Adv.* **8**, eabm3624 (2022)
DOI: 10.1126/sciadv.abm3624

This PDF file includes:

Sections S1 to S7
Figs. S1 to S14
Tables S1 to S3

Other Supplementary Material for this manuscript includes the following:

Table data

S1. MODEL DETAILS

This work utilised two distinct models of COVID-19 disease transmission. One of these is an abstract model, implemented as a branching process to approximate community transmission dynamics. This is applied to quantify the tendency for quarantine breach events to initiate outbreaks, and to characterise those outbreaks. The other model is an agent-based simulation describing disease progression and transmission on the individual level. This is implemented in two scenarios, one used for calibration of fundamental parameters, and the other for investigation of quarantine system efficacy. The calibration scenario is homogeneous, and allows characterisation of the basic reproductive ratio, over-dispersed secondary case rates, and timing of transmission detection events in an effectively open system. The quarantine scenario is designed to reproduce the general features of controlled border screening environments, and implements a defined population structure.

A. Outbreak transmission (Branching process) model

To simulate infectious individuals entering the community and potentially seeding an outbreak, we use a branching process community transmission model with an inhomogenous offspring distribution. Branching process models are considered in terms of generations, and each generation acts independently from the last. We term 'generation zero' to be the index case that leaves quarantine.

1. *Transmission potential of cases in the community*

We define the *transmission potential*, which represents the expected number of cases caused by a single case in the community. The transmission potential combines the biological features of the virus, vaccine status of the index case, vaccination coverage in the wider community and vaccine efficacy parameters.

We break down vaccine efficacy into two components: efficacy against infection, denoted V_I, and efficacy against onward transmission, denoted V_T. The overall reduction in transmission is given by,

$$V = (1 - V_I)(1 - V_T).$$

For a given starting transmission potential, $TP_0 = R_0$, the effective transmission potential in the population, TP_p, is given by,

$$TP_p = TP_0 \cdot (1 - cV),$$

where c is the proportion of the population vaccinated. For a breach caused by an unvaccinated traveller, the first generation transmission potential is given by,

$$TP_u = TP_0 \cdot (1 - cV_I).$$

For a breach caused by a vaccinated traveller, the first generation transmission potential is given by,

$$TP_v = TP_0 \cdot (1 - cV_I)(1 - V_T).$$

2. Secondary case distribution and public health measures

The number of secondary infections generated by a single case is taken from a negative binomial distribution, dispersion $d = 0.1$, and the probability of success being $p = d/(d+TP)$, in order to match the dispersion in the number of secondary cases (fixed at 0.1), and the expected number of secondary cases.

The calendar times at which these infections occur is drawn from a Weibull distribution with mean 5.5 days and standard deviation 1.8, in line with literature estimates [38].

If the case is the index case and thus in quarantine, only infections that occur *after* the individual has left quarantine are retained. If the individual enters isolation at some point, as per worker protocols, then only infections that occur *before* the individual has entered isolation are retained. All future generations are assumed to not be in quarantine or have any proactive isolation applied to them.

3. Probability of a breach becoming an outbreak

To calculate the probability of a single breach event becoming an outbreak, we simulate 10^5 breaches into the community. The index cases for these breaches are chosen randomly from the quarantine model. The probability is calculated separately for travellers and workers.

A breach is defined as an outbreak if it reaches at least five cumulative cases before extinction.

4. Time until significant breach event

The time between breach events are assumed to occur at a frequency defined by the quarantine model. To scale this time according to the number of travellers and the prior probability of arriving to the system infected, we construct a scaling factor,

$$s = \frac{A}{\bar{A}}\frac{I}{\bar{I}},$$

where A is the targeted number of arrival, I is the targeted prevalence of the arrival's country, $\bar{A}$ is the number of arrivals modelled in the quarantine model and $\bar{I}$ is the prevalence of infection assumed in the quarantine model. The time between breaches obtained from the quarantine model is divided by s.

A breach is determined to be an outbreak according to a binomial distribution, with probability of success defined by the probability of causing an outbreak. Traveller and worker breach events are chosen in the same proportion at which they occur from the quarantine model.

B. Individual-based COVID-19 disease model

For the agent-based model of quarantine scenarios, we developed a model of COVID-19 disease and transmission designed to match three salient features:

1. The distribution of delays between symptom onset of a primary case and transmission to secondary cases. Following the definition used by Ferretti et al., we refer to this quantity as Time from Onset of Symptoms to Transmission (TOST) [39].

2. The household secondary attack rate, and secondary case dispersion.

3. The dependence of RT-PCR test sensitivity on time from symptom onset.

Matching these distributions with an individual-based model required the definition a detailed model of disease natural history on the within-host level. The distribution of incubation periods is a key ensemble statistic that informs the model dynamics (see below). There are many possible implementations of within-host models that could generate the required ensemble statistics. Our specific choices follow the logic that virus initially grows exponentially, until recognition by the host immune systems triggers the onset of symptoms (the end of the incubation period). This

immune response produces an exponential decline in viral load until recovery occurs, at which time no replication-competent viral shedding is possible. The sections below detail the specifics of the agent-based model.

1. *The basic reproductive number R_0*

The transmission rate scalar for each individual $\beta_{max} \sim$ Gamma(κ, θ) controls the transmission rate for any given transmission environment. The shape parameter $\kappa = 0.1$ translates directly to the dispersion parameter of the derived secondary case distribution (distributed as a negative binomial, see below). The scale parameter $\theta = \langle\beta_{max}\rangle/\kappa$ is a function of the mean peak force of infection $\langle\beta_{max}\rangle$, which is proportional to R_0 in the calibration model (Figure S1).

To calibrate the basic reproductive number R_0, we performed a systematic scan of $\langle\beta_{max}\rangle$, keeping all other parameters constant. To produce a generic calibration of the basic reproductive number R_0, we performed this scan on an unstructured population with N = 500 individuals, and simulated a large ensemble of single transmission generations without interaction effects (N = 10,000 instances). For each instance, the index case properties are sampled from the parameter distributions specified and transmission is simulated until recovery of the index case. We then count the secondary cases produced, ignoring the additional force of infection produced by secondary cases. The average of these values approximates R_0, and we observe a linear relationship with the control parameter $\langle\beta_{max}\rangle$ (Figure S1). To set the value of R_0 in a given scenario, we use the line of best fit $R_0 = 3.83\langle\beta_{max}\rangle$ to determine the corresponding value of $\langle\beta_{max}\rangle$ required to produce the desired value of R_0.

2. *Secondary case dispersion*

The distribution of secondary cases produced by the index case ensemble used to compute R_0 conforms to a negative binomial distribution with dispersion parameter $r = 0.1$ (Figure S2). This is implemented as follows: for each index case, independent transmission probabilities produce Poisson-distributed secondary case numbers. The transmissibility parameter for each index case β_{max} is sampled from a Gamma distribution, so the secondary case numbers aggregated over all index cases are effectively drawn from an ensemble of Poisson distributions with Gamma-distributed rate parameters. This gives a negative binomial with dispersion $r = \kappa$ [40]. Therefore,

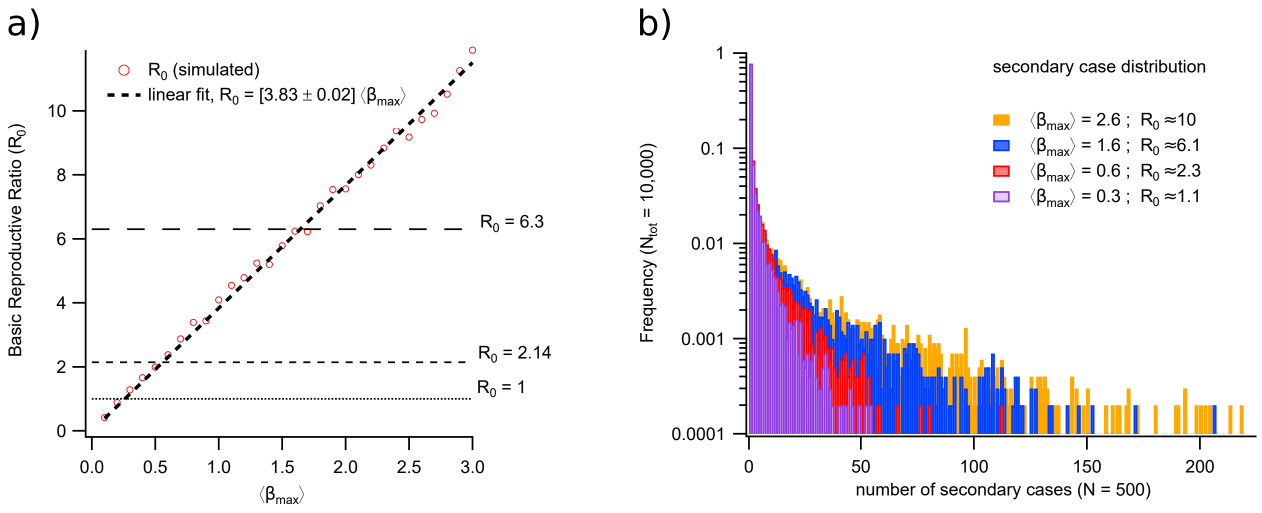

FIG. S1. Calibration of the basic reproductive number R_0. (a) Linear dependence between the global transmission scalar $\langle\beta_{max}\rangle$ and the derived parameter R_0. (b) The distribution of secondary cases produced for different values of R_0, these are effectively drawn from a negative binomial distribution with dispersion parameter ("number of successes" $r = 0.1$), and mean R_0.

the model controls secondary case dispersion directly by assigning β_{max} a Gamma-distributed random variable. The choice of dispersion does not alter the calibration of R_0, which describes only the average number of secondary cases.

3. *Household secondary attack rate*

To check that our parameterisation of R_0 and r produce reasonable correspondence with observed household secondary attack rates, we ran the calibration model setting the population size N = 5. Under these conditions, the number of possible transmissions is akin to a generic number of household contacts (N - 1) and individuals with high transmissibility have restricted transmission potential. This calibration produces average household secondary attack rates in a range between 10% and 25%, consistent with observations of COVID-19 transmission among household contacts (Figure S3) [17, 41]. The low end of the range (10%) is typical for the ancestral lineage of SARS-CoV-2, and this increases consistently with R_0, reaching 25% for $R_0 = 6.3$, corresponding to the Delta variant.

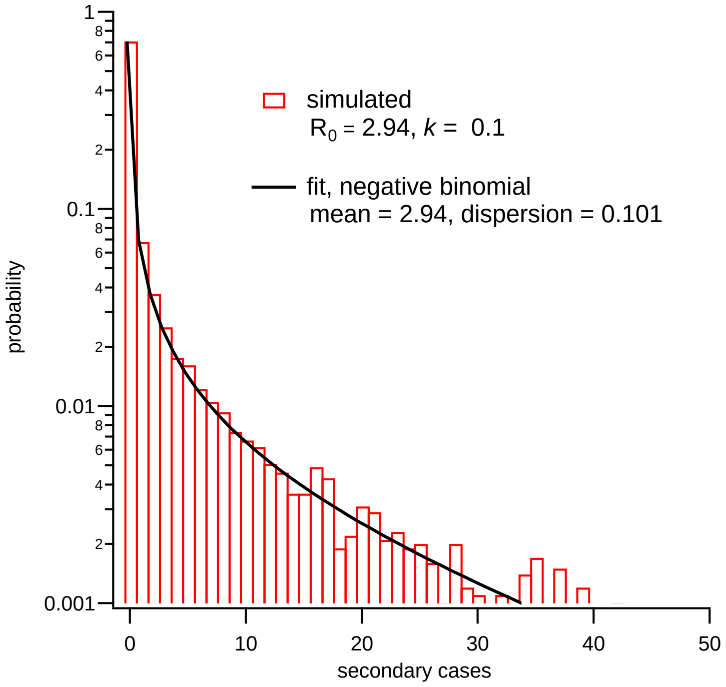

FIG. S2. The secondary case distribution of the calibration model follows a negative binomial with mean R_0. The dispersion parameter (r) is controlled by the shape parameter (κ) of the Gamma distribution from which maximum force of infection (β_{max}) is sampled for each individual.

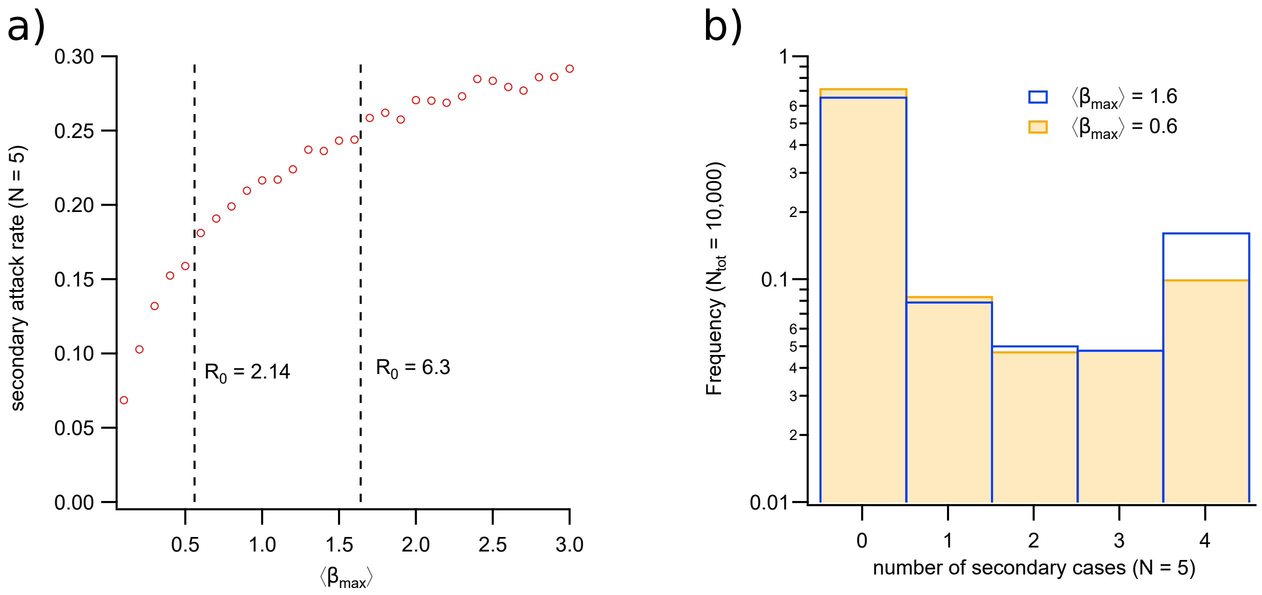

FIG. S3. Secondary attack rate values produced by the calibration model for transmission within small groups (N = 5). (a) Secondary attack rate as a function of the global transmission scalar $\langle\beta_{max}\rangle$. The values plotted correspond to the number of secondary infections produced on average divided by the number of contacts (N - 1 = 4). (b) Frequency distributions of the secondary case numbers produced by 10,000 independent trials each corresponding to a single index case over a single generation of transmission to four close contacts. The values of $\langle\beta_{max}\rangle$ chosen for (b) correspond broadly to estimates of R_0 for the ancestral lineage of SARS-CoV-2 ($\langle\beta_{max}\rangle = 0.6$, $R_0 = 2.14$) and the Delta variant ($\langle\beta_{max}\rangle = 1.6$, $R_0 = 6.3$).

4. Individual-based disease transmission model

Our individual-based model of within-host viral dynamics follows a three-part function, the precise form of which is determined by each individual's incubation period. An individual trajectory of infectiousness as a function of time from exposure $\beta(t)$ is illustrated in Figure S4. This function features an initial exponential increase, followed by a brief plateau and subsequent exponential decline. The start of the exponential decline phase corresponds to the end of individual's incubation period (onset of symptoms). Note that individuals become infectious immediately after they are infected, with no latent period preceding the onset of infectiousness. In our model, we assume that 1/3 of infected individuals never develop symptoms, which reduces the probability they will be detected within the quarantine system. In our model we use the same functional form of $\beta(t)$ to describe all infections, regardless of whether or not they are asymptomatic. It may be that asymptomatic individuals are marginally less contagious, in which case our model would overestimate the number of secondary cases they produce [42]. In this work, we opted for the conservative assumption that no difference exists.

The within-host dynamics are implemented as a piecewise function, with a plateau between growth and decay phases describing $\beta(t)$, the time-dependent infectiousness of an individual:

$$\beta(t) = \begin{cases} \frac{\beta_{\mathsf{max}}}{V_{\mathsf{max}}}\big[\exp(k_1 t) - 1\big] & t \leq t_{\mathsf{inc}} - T_p \\ \beta_{\mathsf{max}} & t_{\mathsf{inc}} - T_p \leq t < t_{\mathsf{inc}} \\ \beta_{\mathsf{max}}\big[\exp\big(k_2[t - t_{\mathsf{inc}}]\big) - [V_{\mathsf{max}}]^{-1}\big] & t \geq t_{\mathsf{inc}} \end{cases}, \tag{S1}$$

where t_{inc} is the incubation period of the individual, T_p is the duration of the infectiousness plateau (set equal to $0.1t_{\mathsf{inc}}$), and β_{max} is the maximum infectiousness of that individual. The parameter V_{max} is a scaling factor controlling the shape of the growth curve (smaller values of V_{max} produce broader growth and decay functions, higher values produce steeper growth and decay). The rate parameters k_1 and k_2 are determined by the value of V_{max}, and the duration of incubation and post-incubation periods:

$$k_1 = \ln(V_{\mathsf{max}})\ [t_{\mathsf{inc}} - T_p]^{-1}, \tag{S2}$$

and

$$k_2 = \ln\left([V_{\mathsf{max}}]^{-1}\right)\ t_{\mathsf{r}}^{-1}, \tag{S3}$$

where t_r is the time between symptom onset and recovery, which is drawn uniformly at random from the range [5d, 10d], to approximately match the duration of replication-competent viral shedding after symptom onset [43]. For an interaction between infected individual i and susceptible individual j, the probability of transmission is computed as:

$$p_{ij}(t) = 1 - \exp(-\beta(t,i)\sigma_{ij}\Delta t)\,, \tag{S4}$$

where $\beta(t,i)$ is the force of infection produced by an infected individual i at time t since infection, $\Delta t = 0.1$ days is the duration of a discrete time step, and σ_{ij} is a scaling factor that incorporates the effects of contact frequency and intensity for a given transmission event between infected individual i and susceptible individual j:

$$\sigma_{ij} = F_{ij} h_j^{-1}\,, \tag{S5}$$

where F_{ij} is a context-specific transmission mitigation factor, and h_j is the number of contacts of a given type in the local mixing environment. In the calibration model, $F_{ij} = 1$ and $h_j = N - 1$. In the full quarantine model, F_{ij} is given as follows:

- For close contacts between travellers in the same group,
  $F_{ij} = 1$, and $h_j = n_i - 1$, where n_i is the number of close contacts in the same group as individual i ($n = 4$ initially but can decrease if members are moved into isolation).

- For interactions between travellers in different close contact groups,
  $F_{ij} = 0.01$ and $h_j = \sum\limits_{g|i \notin g} n_g$ is the number of travellers in the quarantine system who are not isolated and who are not in the same group g as individual i).

- For interactions between infected travellers and susceptible workers,
  $F_{ij} = 0.01$ and $h_j = n_w$, where n_w is the number of workers *present* in the facility at time t.

- For interactions between infected workers and susceptible travellers,
  $F_{ij} = 0.01$, $h_j = n_{tot}$ where n_{tot} is the number of travellers not in isolation.

- For interactions between infected workers and susceptible workers,
  $F_{ij} = 0.1$ and $h_j = n_w - 1$, where n_w is the number of workers *present* in the facility at time t.

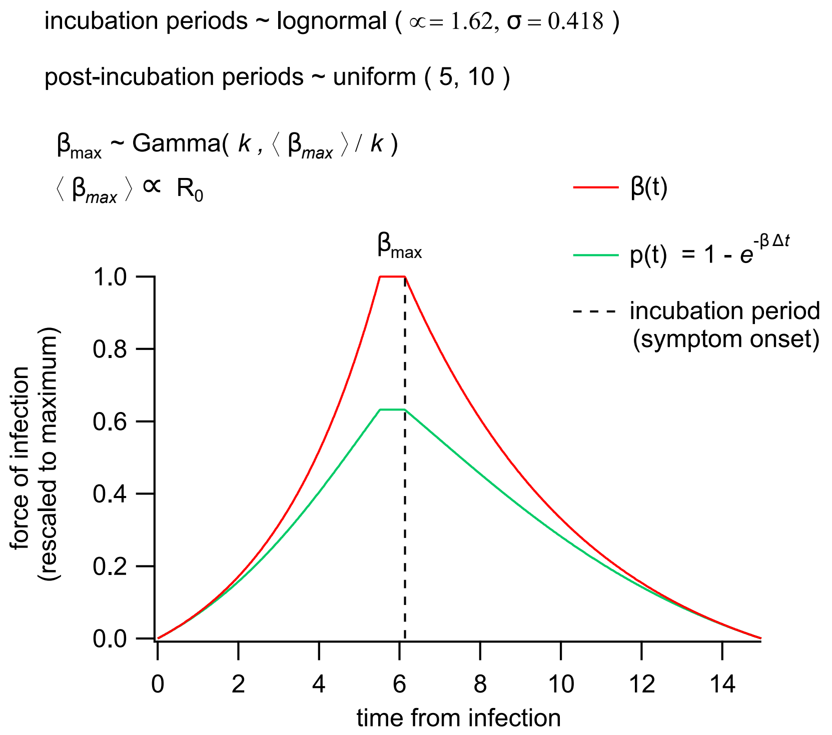

FIG. S4. An example of the force of infection produced by an infected individual as a function of time from infection. The force of infection increases exponentially from the time of infection to the start of the plateau, at which point it reaches its maximum value. The plateau phase lasts until the end of the incubation period and has a duration equal to 1/10 of the total incubation period. Incubation periods are drawn from a log-normal distribution. During the post-incubation period, infectiousness decreases exponentially until it reaches a cutoff at the time of recovery. The duration between the end of the plateau phase and recovery is drawn from a uniform distribution bounded between 5 and 10 days. The y-axis values are re-scaled to β_{max}, which is drawn from a Gamma distribution parameterised for a specified R_0 value as described in Section S1 B 1.

5. Time from Onset of Symptoms to Transmission (TOST)

The functional form used for $\beta(t)$ was developed to match the ensemble distributions of time from onset of symptoms to transmission (TOST), reported by Ferretti et al. [39]. A comparison of the statistics produced by the calibration model and the distribution reported by Ferretti et al. is shown in Figure S5. The qualitative match between our model's case statistics and the empirical TOST distribution is sensitive to the choice of individual disease trajectory function

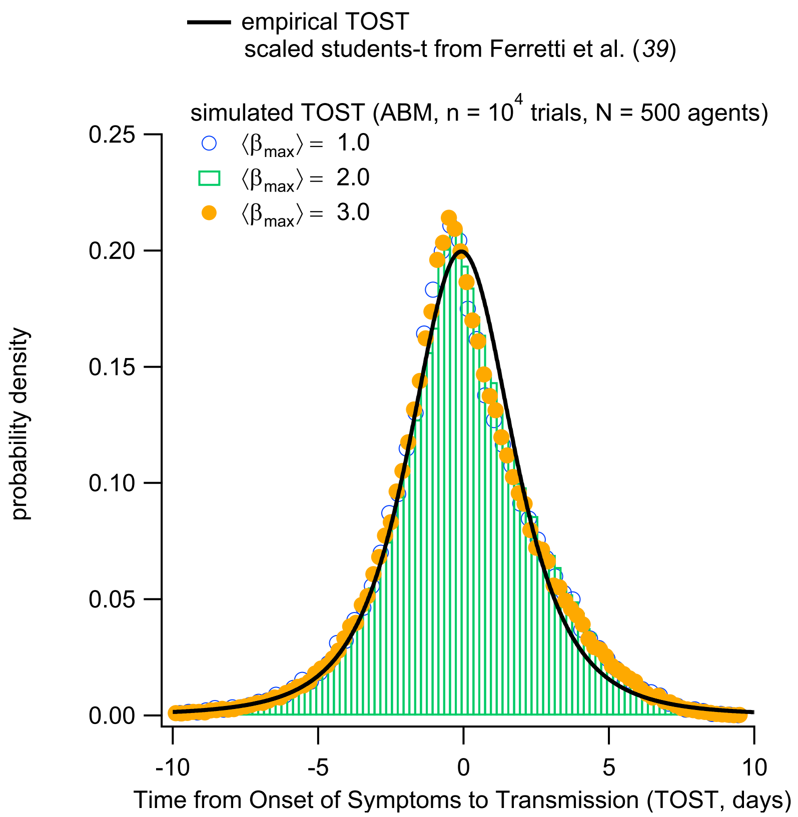

FIG. S5. Time from Onset of Symptoms to Transmission (TOST) distributions produced by the calibration model with N = 500 individuals in a fully-connected (homogeneous) contact network. The TOST distribution does not depend on the transmissibility of the disease, and matches closely with the best-fit model from Ferretti et al. [39].

(Equations S1 through S3).

6. *Time from Onset of Symptoms to Detection (TOSD)*

The model of test sensitivity discussed in the following subsections is designed to capture the variability in RT-PCR test sensitivity as a function of time relative to symptom onset. These models are based on a study by Hellewell et al. [36]. Here, the general objective is to describe test sensitivity as a piecewise sigmoid with a single breakpoint, T_c. Before the breakpoint sensitivity increases ($t < T_c$, where t represents time from infection). After the breakpoint ($t > T_c$), sensitivity decreases slowly. In this case, the function is expressed as a logistic regression:

parameter	95% CI
T_c	[2.01, 5.11]
b_1	[0.8, 2.31]
b_2	[1.26, 3.47]
b_3	[-1.2, -1.05]

TABLE S1. Parameter ranges used in Hellewell et al. [36], to describe the progression of test sensitivity during COVID-19 infections in healthcare workers. In the model reported here, these parameter ranges were adjusted to account for individual variability in incubation periods.

$$p(\text{positive}|\text{infected}) = \begin{cases} [1+\exp(-(b_1+b_2\tau))]^{-1} & t \le T_c \\ [1+\exp(-(b_1+b_2\tau+b_2b_3\tau)]^{-1} & t > T_c \end{cases}, \tag{S6}$$

where $\tau = t - T_c$, the maximum test sensitivity depends only on b_1 as $[1+\exp(-b_1)]^{-1}$, the initial growth of sensitivity is controlled by b_2, and the decay of sensitivity after T_c is given by b_2 and b_3. The parameter ranges (95% credible intervals) provided in Hellewell et al. are shown in table S1.

The parameter ranges shown in Table S1 were produced by fitting to ensemble data. That is, the study examined a timeseries of tests and test results for a cohort of 27 individuals, and aggregated this timeseries in order to fit the parameters of the model. Therefore, the parameter ranges above are not suitable for all individual test sensitivity trajectories. Our individual-based model translates the specified functional form (piece-wise logistic function) and parameter ranges into a set of trajectories describing test sensitivity as a function of the incubation period (time between infection and symptom onset) for each individual in the system. By doing so, we reconcile the ensemble characteristics observed by Hellewell et al., with the requirements of individual trajectories. In our model, the requirements are specified as follows:

- An individual may not test positive before they are infected.

- The peak of test sensitivity must have some correspondence to the timing of the peak viral load (peak infectiousness).

- The probability of detection should be high during the first week of symptoms.

These requirements have the following implications for the model framework specified by Hellewell et al.:

- the specified range of T_c will not fit trajectories with longer-than-average incubation periods (average incubation period is 5.5 days).

- The rate of increase (b_2) must be higher for individuals with shorter incubation periods (this imposes a correlation when specifying individual trajectories).

To address these discrepancies, we relax the parameter restrictions in Table S1, and set the breakpoint position relative to an individual's incubation period. The delay between the breakpoint T_c and the onset of symptoms t_{inc} is distributed in the range $[0, 4.11]$.The rationale for this choice is based on the observation that the upper 95% credible interval reported by Hellewell, $T_c = 5.11$, is approximately equivalent to the median incubation period. Here, we infer that this corresponds to using the incubation period as an upper limit for T_c. Additionally, our model correlates the length of the delay with the incubation period of each agent by quantile matching, to ensure the that T_c is always positive:

$$T_{c,i} = t_{inc,\ i} - 4.11 q_i \,, \tag{S7}$$

where i denotes a specific individual, q_i is the value of the incubation period CDF evaluated at $t_{inc}(i)$, and 4.11 is the range of possible delays (in units of days) between peak test sensitivity and symptom onset.

To provide a better match to ensemble statistics, we also chose to impose a negative correlation between individual incubation periods and the term (b_2) that specifies the growth rate of test sensitivity before the peak, and (along with b_3) the decay rate of test sensitivity after the peak:

$$b_{2,i} = 1.26 - 2.21(1 - q_i) \,, \tag{S8}$$

where 1.26 is the lower bound for b_2 and 2.21 is the range of b_2 values given in Table S2. The modified parameter ranges are shown in Table S2, samples of individual trajectories are shown in Figure S6, and a comparison of ensemble statistics is shown in Figure S7. On the other hand,

parameter	95% CI
T_c	t_{inc} - [0, 4.11]
b_1	[0.8, 2.31]
b_2	[1.26, 3.47]
b_3	[-1.14, -1.05]

TABLE S2. Parameter ranges used in order to fit the ABM ensemble statistics to those reported by Hellewell et al. [36].

the b_1 and b_3 parameters are chosen uniformly at random for each individual from the ranges specified in $S2$.

The model as-implemented approximately matches the proportion of infections detected before symptom onset, given daily RT-PCR test, estimated by Hellewell et al. [36] (approx. 75%). The corresponding distribution of time from onset of symptoms to detection (TOSD), is shown in Figure S7(c). In this system, daily testing detects approximately 78% of cases prior to symptom onset.

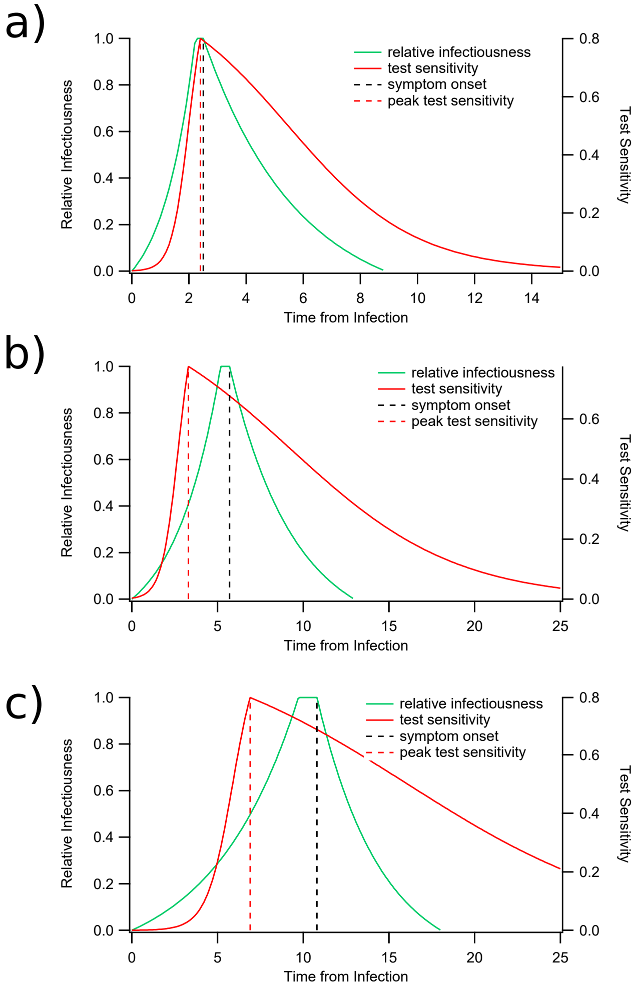

FIG. S6. Individual trajectories of test sensitivity and force of infection as a function of time since exposure. (a) Short incubation periods produce alignment of peak test sensitivity and peak infectiousness, both of which occur close to the onset of symptoms. (b) Average incubation periods produce a test sensitivity peak that lags symptom onset and peak infectiousness by several days, and extend the tail of the test sensitivity curve after symptom onset. (c) Long incubation periods correspond to longer lag times between peak test sensitivity and symptom onset, and further extend the tail of the test sensitivity trajectory.

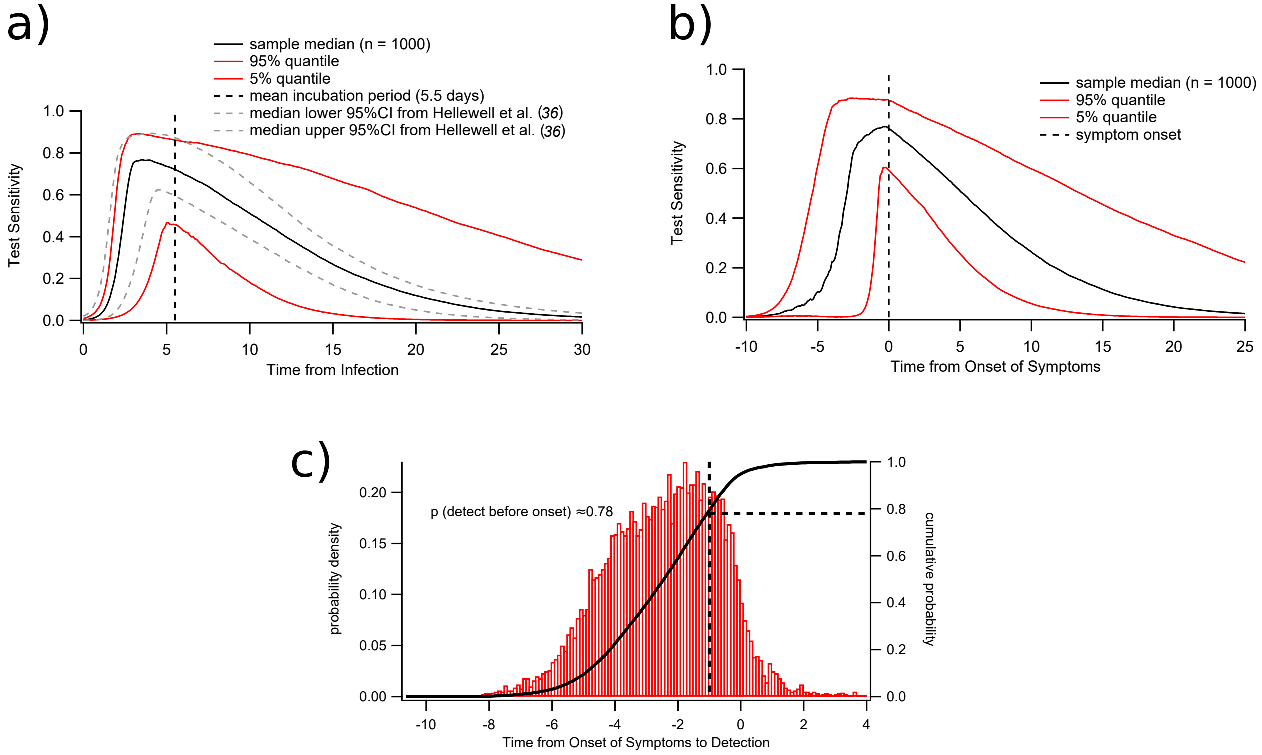

FIG. S7. Ensemble statistics of test sensitivity as a function of time from infection, aggregated from the Agent based model. (a) Test sensitivity as a function of time from infection. Grey dashed lines give the 95% credible intervals of the mean from Hellewell et al., while the solid black trace shows the ensemble average produced by 1000 samples from the Agent-based model. Solid red traces give the 5% and 95% quantiles from the Agent-based model. (b) Test sensitivity as a function of time from symptom onset produced by the Agent-based model. The black trace gives the ensemble average while the red traces give 5% and 95% quantiles. The vertical dashed lines in (a) and (b) represent the onset of symptoms. (c) Time from Onset of Symptoms to Detection given daily tests via RT-PCR, derived from the Agent-based model. The solid trace indicates the cumulative distribution, while the dashed lines indicate the proportion of cases (0.78) detected more than one day prior to symptom onset (assuming a test turnaround of 1 day, this corresponds to the proportion detected prior to symptom onset).

C. Model of quarantine environment

The model of quarantine system in which transmission occurs, explicitly represents individual travellers and quarantine workers. We describe the model in terms of an Input layer, Filter layer and Output layer.

1. Input layer

Arriving travellers are characterised in terms of:

- the proportion of arriving travellers who are infected
- the proportion of arriving travellers who are vaccinated

Here, we set the proportion of infected arrivals to 1% and the proportion vaccinated to 100%. Additionally, it is assumed that infected arrivals are either pre-symptomatic or asymptomatic. If an arrival is pre-symptomatic their time since infection is sampled at random from the individual's incubation period. If a traveller is asymptomatic, their time since infection on arrival is sampled from the sum of their incubation period and post-incubation period.

2. Filter layer

Travellers: Quarantined travellers are structured into groups who are quarantined together (e.g., in the same room). For the results presented here, the size of these groups is set to 4, and the number of travellers in the facility is set to 100. Travellers are discharged from the system in groups. Once all travellers in a group meet the discharge criteria, the group is removed and a new group generated to replace them.

Workforce: The quarantine workforce is also represented. The workforce model specifies a weekly work schedule for each individual, including 5 days working and 2 days off. On days and off days are scheduled randomly, and a work schedule configuration is accepted by the model as long as at least 5/20 workers are present each day of the week.

Transmission control: We assume that the rate of transmission between travellers who belong to different groups is reduced by a factor of 100, compared to that between travellers

who belong to the same group. Similarly, we assume a factor of 100 reduction in transmission between travellers and workers, and a factor of 10 reduction between workers.

Vaccination: The efficacy of vaccination may be varied to reflect available evidence. As above, we assume that some proportion of the workforce, and some proportion of arriving travellers will have been vaccinated. Vaccine efficacy parameters can be varied to reflect the characteristics of vaccines used in particular source countries of interest. In this work, we assume that all individuals in the quarantine system are vaccinated. We implement vaccination by setting efficacy against infection ($V_I = VE$) and efficacy against onward transmission $V_T = 0$. This choice is not significant when computing transmission within the quarantine system, because all individuals are assumed vaccinated. However, the components of vaccine efficacy could play a significant role when interpreting the force of infection produced by breach events (see sensitivity analysis below).

Testing: Testing of workers can be scheduled at varying frequencies (e.g., daily, every three days, or weekly) and workers will only be tested on days that they attend the workplace. In the results reported here, testing of the workforce is performed daily, as long as a worker is present.

The model includes testing of travellers that can be scheduled to occur on given days during their quarantine period. For the results reported here, travellers were tested on days 3 and 12 after entering quarantine. We calibrated test sensitivity as specified above, to represent detection via RT-PCR.

Response to a positive test or symptoms: If a worker tests positive or develops symptoms, we assume that they are removed from the workforce and replaced by a new worker (irrespective of whether they attend work that day or not).

If a traveller tests positive or develops symptoms, they are isolated and removed from the quarantine facility (e.g., to a "health hotel" or hospital), the isolation period is set to 10 days, after which they are released. A positive test result in a traveller triggers a 14-day quarantine extension for other travellers in the same group, and resets their testing schedule (tests performed on days 3 and 12 after extension).

3. Output

The output of the quarantine simulation is a time series of "breach events" with the corresponding characteristics of each individual that leaves quarantine while infected. Several properties are recorded, and the recorded properties depend on whether the infected individual was a worker or traveller. For travellers, the recorded properties are:

- *exposure days*: the number of days they will remain infectious
- *days in quarantine*: the total number of days spent in the quarantine system
- *days in extended quarantine*: the number of days in quarantine extension after detection of a case in a close contact
- *days in isolation*: the number of days spent in isolation after testing positive or presenting symptoms
- *incubation period*: the period between infection and symptom onset
- *time post-incubation*: the period between symptom onset and recovery
- *time discharged*: the timepoint at which the individual was released from quarantine
- *index case (boolean)*: a flag indicating whether the individual arrived infected or was infected while in quarantine
- *symptomatic (boolean)*: a flag indicating whether the individual would express clinical symptoms after their incubation period
- *vaccinated (boolean)*: a flag indicting the individual's vaccination status
- β_{max}: the maximum force of infection for the individual (i.e., force of infection at symptom onset)
- $\beta_{community}$: the integrated force of infection over the period after the individual was discharged from quarantine (these are summed to produce the β_{tot} value for a simulation)

For workers, the recorded properties are:

- *exposure days*: the number of days between infection and detection or recovery
- *incubation period*: same as for travellers (see above)
- *post-incubation period*: same as for travellers (see above)
- *tested positive (boolean)*: a flag indicating whether the individual was removed after testing positive
- *expressed symptoms (boolean)*: a flag indicating whether the individual was removed after expressing symptoms
- *time discharged*: the timepoint at which the individual was removed from the facility
- *symptomatic (boolean)*: same as for travellers (see above)
- *vaccinated (boolean)*: same as for travellers (see above)
- β_{max}: same as for travellers (see above)
- $\beta_{community}$: the force of infection integrated over the period from infection to detection and discharge.

This output timeseries is used to generate input statistics for the index cases used in the branching process model.

S2. SENSITIVITY ANALYSIS OF INCUBATION PERIOD

To estimate the effect of a shorter incubation period, we used the statistics reported by the China CDC for an outbreak of the Delta variant in May, 2021 [16]. While their estimates of the incubation period may differ from those of others [17], they represent a lower bound for this important parameter. Here, we demonstrate how a shorter incubation period affects the transmission and detection of the virus in our model system. The main differences are:

- Without intervention, slightly less transmission occurs prior to symptom onset.
- Due to a more rapid increase in viral load, detection of cases typically occurs earlier.

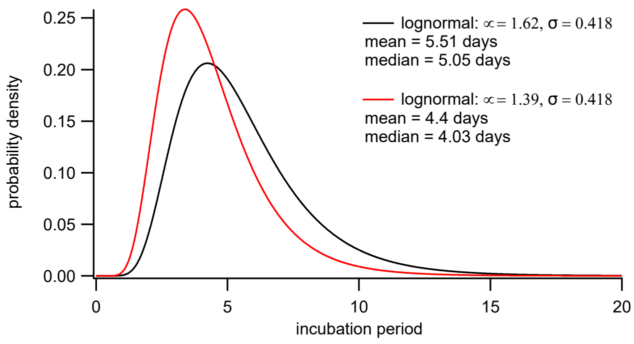

FIG. S8. Incubation period distributions used in the Agent-based model. The black trace indicates the distribution used for the main results, while the red trace indicates the distribution used in the sensitivity analysis.

- In the quarantine model scenario, a shorter overall duration of infection increases the efficacy of a 14-day quarantine.

These results demonstrate how shorter incubation periods can correspond to an enhanced capacity to control transmission. Our results caution against the use of these preliminary estimates of incubation period statistics for use in models of border quarantine systems. As we demonstrate, they potentially over-estimate the effectiveness of these systems and do not represent conservative assumptions about viral dynamics.

To investigate the effect of incubation period, we first re-calibrated the base model using the modified incubation period distribution (Figure S9). During calibration, we determined the timing of symptom onset relative to transmission, and the timing of detection relative to symptom onset, given daily testing via RT-PCR in a homogeneous transmission network of 500 individuals (Figure S10). We then applied this modified calibration in the full quarantine simulation to examine the effectiveness of quarantine over the $VE \times R_0$ parameter space. The results are qualitatively similar to those produced by the original calibration (average incubation period 5.2 days), but show consistently lower levels of breach risk as measured by β_{tot}.

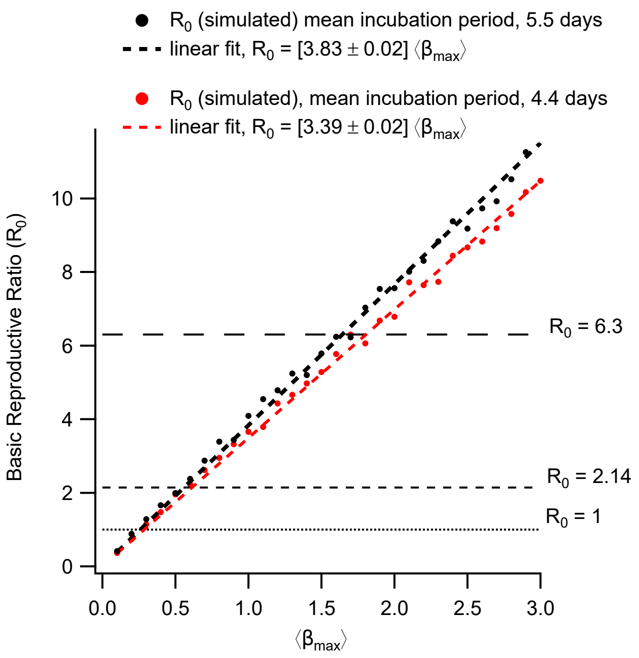

FIG. S9. Calibration of R_0 as a function of the global transmission scalar for two different incubation periods.

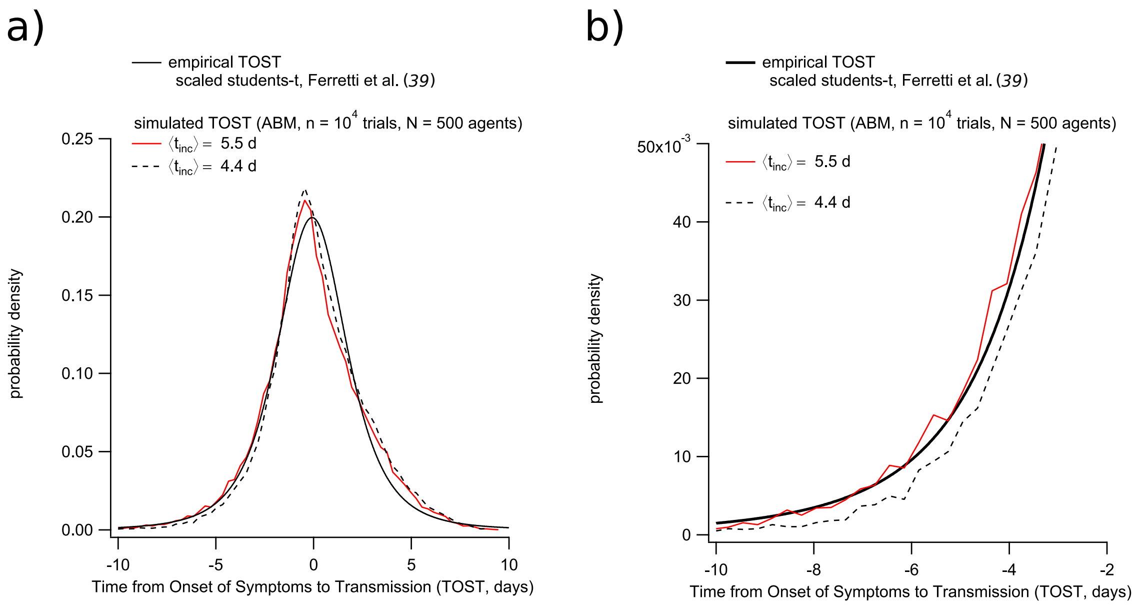

FIG. S10. Distribution of TOST for two different incubation period distributions. (a) Shows the full distribution, while (b) zooms in on the negative intervals corresponding to pre-symptomatic transmission.

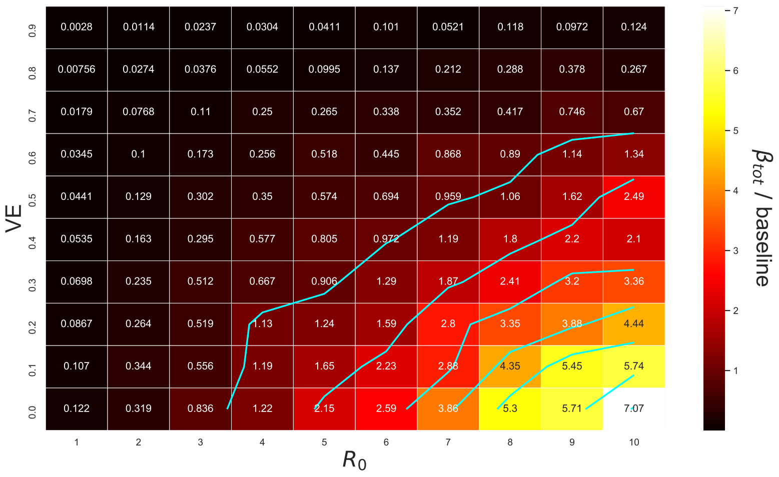

FIG. S11. Integrated force of infection (β_{tot} relative to baseline) using an incubation period with a mean of 4.4 days. The baseline (denominator) value is taken from the alternate scenario with an incubation period of 5.5 days ($R_0 = 3, VE = 0$). Results are shown as a function of vaccine efficacy and R_0.

S3. SENSITIVITY ANALYSIS OF VACCINE EFFICACY AGAINST ONWARD TRANSMISSION

For the results presented in Figure 3, it was assumed that vaccine efficacy against onward transmission is negligible. That is, the values reported for β_{tot} represent differences in breach statistics rather than the direct effects of vaccine efficacy. Here, we examine the alternate extreme case in which efficacy against transmission is equivalent to VE. This equates to multiplication of the β_{tot} values in Figure 3 by the factor $1 - VE$. Doing so results in a correction applied to the force of infection values, which fall linearly as VE increases. Under this alternate assumption, for each value of R_0 the vaccine efficacy required to maintain baseline outbreak statistics is lower (S12). The magnitude of the difference increases with R_0, ranging from approximately 10% (for $R_0 = 4$) up to approximately 20% (for $R_0 = 10$). While these differences are substantial, they represent the largest possible deviation due to efficacy against transmission. Therefore, the results presented in Figure 3 may overestimate the vaccine efficacy required to maintain baseline risk ratios, but the magnitude of this overestimate cannot exceed 20%.

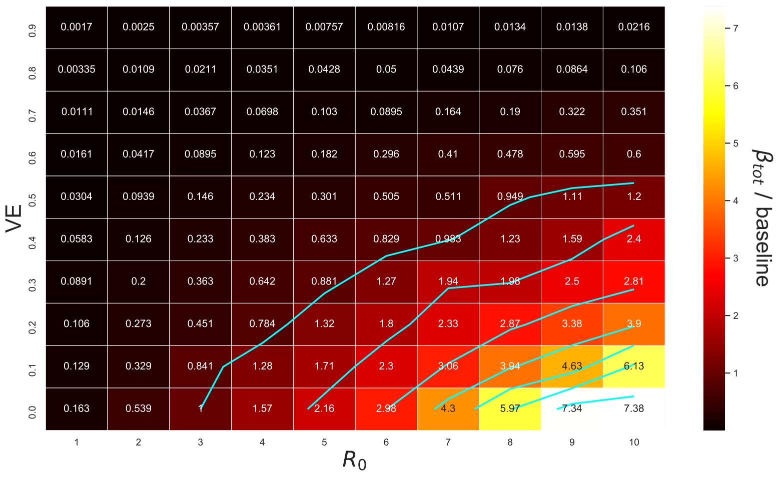

FIG. S12. Integrated force of infection (β_{tot} relative to baseline) assuming vaccine efficacy against onward transmission is maximised ($V_T = VE$, $V_I = 0$). The baseline (denominator) value is taken from the scenario with $R_0 = 3$, and $VE = 0$. Results are shown as a function of vaccine efficacy and R_0.

S4. SENSITIVITY ANALYSIS OF SYMPTOMATIC FRACTION

To investigate the sensitivity of our results to the choice of symptomatic fraction, we repeated the parameter sweep over R_0 and VE (as shown in Figure 3), for three alternate values of the symptomatic fraction (Figure S13). We chose to examine the two most extreme assumptions, in which either all (Figure S13a) or none (Figure S13c) of the infected cases express symptoms, as well as one additional intermediate case in which half of infections are symptomatic (Figure S13b). If the symptomatic fraction is allowed to decrease with VE (Figure S13d), the vaccine efficacy required to maintain baseline risk increases marginally, but the risk associated with low VE and high R_0 resembles the base scenario (low VE is compensated for by higher frequency of symptom expression).

S5. SENSITIVITY ANALYSIS: LIMITED INFECTION CONTROL

Here, we demonstrate how the quarantine system model performs when infection control is assumed to be less effective by a factor of 10. This means that transmission rates between

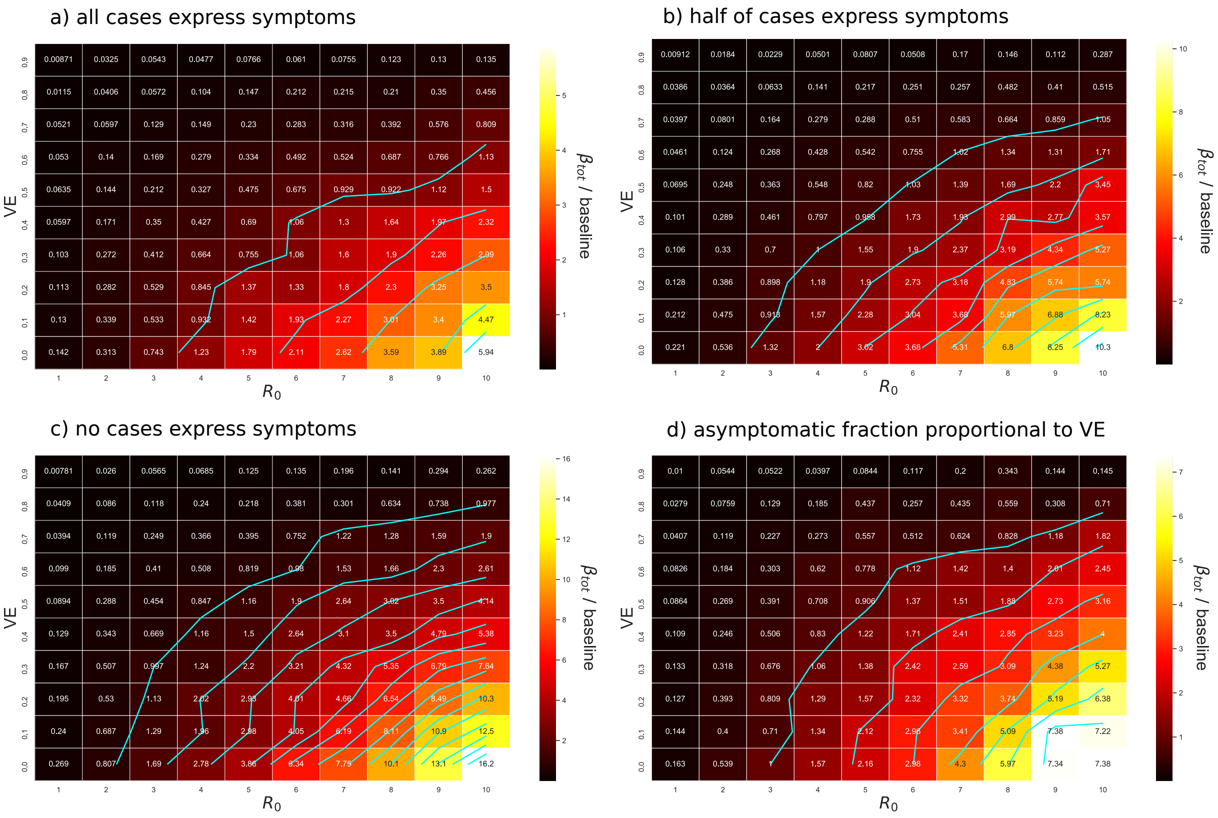

FIG. S13. Integrated force of infection (β_{tot} relative to baseline) assuming different probability of symptom expression. (a) If all cases express symptoms, community infections are reduced, relative to what is reported in Figure 3. However, if the symptomatic fraction is drops to 0.5 (b) or 0 (c), community cases increase up to approximately a factor of two in the extreme that no cases are detected through symptom expression. In (d), the symptomatic fraction is computed as $p_{symp} = 0.67(1 - VE)$, which marginally increases the vaccine efficacy required to maintain baseline risk with increasing R_0, relative to the core scenario with constant $p_{symp} = 0.67$.

travellers in different groups are reduced by a factor of 10. Likewise, transmission from travellers to workers is reduced by a factor of 10. However, transmission between workers is unmitigated. The results, in terms of the integrated force of infection relative to baseline, are shown in Figure S14.

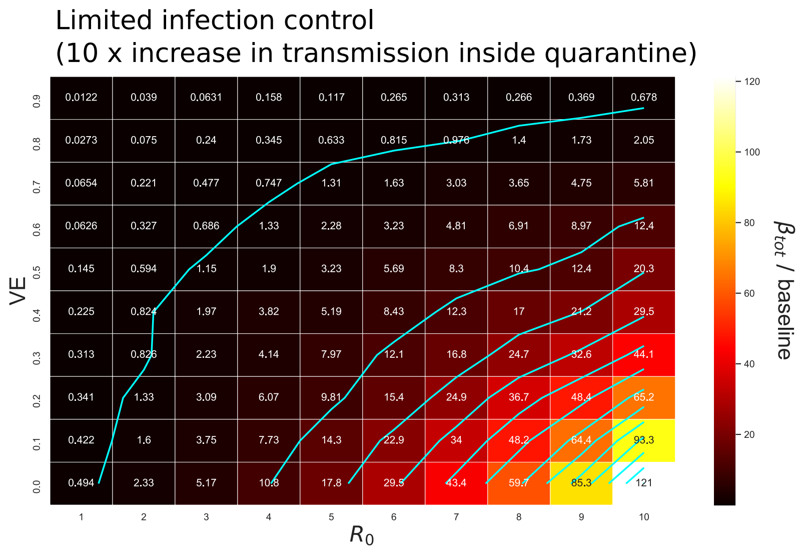

FIG. S14. Integrated force of infection (β_{tot} relative to baseline) assuming the effectiveness of infection control within quarantine is reduced by a factor of 10. The baseline (denominator) value is taken from the scenario with $R_0 = 3$, and $VE = 0$, using the infection control measures assumed in the base model. Results are shown as a function of vaccine efficacy and R_0. Note that the contour bands show increments corresponding to factors of 10 relative to baseline.

S6. BREAKDOWN OF BREACH EVENTS BY TYPE

Here, we show tables corresponding to the scenarios that produced the histograms in Figure 5. Table S6 categorise breach events into eight categories, to differentiate between primary and secondary cases, and to stratify the model output by the quarantine pathway (i.e., whether or not the infected individual was isolated or put into extended quarantine before release). These tables demonstrate a few important points about the function of the quarantine system. First, they show that high breach event counts do not necessarily correspond to high community exposure levels, because cases that leave the system near the end of their infection are not as infectious and present a dramatically reduced risk. Second, they demonstrate the two primary roles of vaccination: (1) reducing the number of infected primary cases entering the system and (2) reducing transmission to secondary cases within the system.

R0 = 3, VE = 0					
case type	isolated?	quarantine extended?	n events	exposure days	integrated force of infection
primary	yes	yes	19	25.9	21.41
		no	2638	3120.1	419.18
	no	yes	0	0	0.00
		no	146	480.2	20.74
secondary	yes	yes	1678	2232.5	277.99
		no	624	874.3	191.15
	no	yes	466	2228.3	359.46
		no	322	3085.1	859.82

mitigated risk	< 10
expected risk	10 - 100
partial failure	101 - 500
total failure	> 501

R0 = 3, VE = 0.9					
case type	isolated?	quarantine extended?	n events	exposure days	integrated force of infection
primary	yes	yes	1	1.8	0.16
		no	252	329.5	51.92
	no	yes	0	0	0.00
		no	14	46.4	0.99
secondary	yes	yes	30	38.7	4.00
		no	9	15.1	1.79
	no	yes	10	51.9	29.39
		no	4	21.3	2.97

R0 = 6, VE = 0					
case type	isolated?	quarantine extended?	n events	exposure days	integrated force of infection
primary	yes	yes	31	28.1	36.59
		no	2649	3326.9	745.56
	no	yes	4	3.8	4.81
		no	144	536.6	26.48
secondary	yes	yes	2522	3267.1	974.24
		no	1047	1495.8	334.42
	no	yes	637	3001.4	1100.20
		no	581	5816.2	2294.72

R0 = 6, VE = 0.8					
case type	isolated?	quarantine extended?	n events	exposure days	integrated force of infection
primary	yes	yes	0	0	0.00
		no	540	634.2	189.30
	no	yes	0	0	0.00
		no	33	96.9	6.23
secondary	yes	yes	205	286	83.30
		no	70	89.9	16.16
	no	yes	46	255.9	137.38
		no	28	243.3	154.35

TABLE S3. Detailed breakdown of breach events and the associated days of community exposure (exposure days) and integrated force of infection produced by each of eight possible pathways in four illustrative scenarios using different values of R_0 and VE. Note that the integrated force of infection shown was computed assuming no effect of the vaccine on the capacity to infect others.

S7. LIST OF DOCUMENTED QUARANTINE BREACH EVENTS

The online supplementary material includes a copy (abm3624_Suppl. Excel_seq1_v1.xlsx) of the Australian managed quarantine breach event statistics as reported on the website `covidlive.com.au`. The enclosed list of quarantine breach events was complied from goverment reports and media releases and was downloaded by the authors of this work on December 20th, 2021.
